# Factors Influencing Tobacco Smoking and Cessation Among People Living with HIV: A Systematic Review and Meta-analysis

**DOI:** 10.1007/s10461-024-04279-1

**Published:** 2024-03-13

**Authors:** Thanh H. L. Hoang, Van M. Nguyen, Louise Adermark, Gloria G. Alvarez, Donna Shelley, Nawi Ng

**Affiliations:** 1 School of Public Health and Community Medicine, Institute for Medicine, Sahlgrenska Academy, University of Gothenburg, Gothenburg, Sweden; 2 Department of Pharmacology, Institute of Neuroscience and Physiology, Sahlgrenska Academy, University of Gothenburg, Gothenburg, Sweden; 3 School of Global Public Health, New York University, New York, USA; 4 School of Public Health and Community Medicine, Institute for Medicine, Sahlgrenska Academy, University of Gothenburg, Medicinaregatan 18A, 41390 Gothenburg, Sweden

**Keywords:** Tobacco smoking, Smoking cessation, HIV, Systematic review, Meta-analysis, Substance use

## Abstract

Tobacco smoking is highly prevalent among people living with HIV (PLWH), yet there is a lack of data on smoking behaviours and effective treatments in this population. Understanding factors influencing tobacco smoking and cessation is crucial to guide the design of effective interventions. This systematic review and meta-analysis of studies conducted in both high-income (HICs) and low- and middle-income countries (LMICs) synthesised existing evidence on associated factors of smoking and cessation behaviour among PLWH. Male gender, substance use, and loneliness were positively associated with current smoking and negatively associated with smoking abstinence. The association of depression with current smoking and lower abstinence rates were observed only in HICs. The review did not identify randomised controlled trials conducted in LMICs. Findings indicate the need to integrate smoking cessation interventions with mental health and substance use services, provide greater social support, and address other comorbid conditions as part of a comprehensive approach to treating tobacco use in this population. Consistent support from health providers trained to provide advice and treatment options is also an important component of treatment for PLWH engaged in care, especially in LMICs.

**Supplementary Information:**

The online version contains supplementary material available at 10.1007/s10461-024-04279-1.

## Introduction

Tobacco use is substantially greater in people living with human immunodeficiency virus (HIV) (PLWH), compared to the general population [1]. The double burden of tobacco smoking and HIV transmission is particularly high in low-resource countries [2–5]. Although improved access to antiretroviral therapy (ART) has significantly reduced HIV-related morbidity and mortality, tobacco smoking threatens to diminish those gains [6–12]. Compared to PLWH who do not use tobacco, PLWH who smoke have higher rates of tobacco- and HIV-related diseases and poorer adherence and treatment response to ART [13–15]. Besides, AIDS-related deaths are higher in smokers living with HIV than in their non-smoking counterparts, resulting in the difference in life expectancy between these two groups of about 12.3 years [16]. Given the high prevalence of tobacco smoking and its detrimental health effects on PLWH, promoting smoking cessation is essential to address this modifiable risk factor, especially among populations in low- and middle-income countries (LMICs) where the burden is heavier, and the gaps in the literature on effective interventions to address tobacco smoking among PLWH are greater [2, 17, 18].

Despite the availability of evidence-based smoking cessation interventions targeting PLWH, many intervention components are not tailored to the unique needs of PLWH to maintain long-term smoking abstinence [18]. Furthermore, studies have shown that compared to the general population, PLWH had lower quit rates and readiness to quit, which were associated with drug abuse, greater emotional issues, and fewer quit attempts [19, 20]. Many studies have identified characteristics of smoking PLWH and determinants of their quitting behaviour. However, no existing systematic review has attempted to scrutinise the associated factors of tobacco smoking and smoking cessation of PLWH to inform future interventions.

A thorough understanding of the demographic, social, behavioural, and cultural factors that affect smoking and cessation behaviour of PLWH is crucial to determine appropriate approaches to reduce tobacco use among this population. Therefore, we conducted a systematic review to synthesise and meta-analyse factors influencing smoking and cessation behaviours, including current tobacco smoking and smoking cessation among PLWH. The differences in associated factors between high-income countries (HICs) and LMICs were also examined in our sub-analyses to understand the unique needs of PLWH in the two settings.

## Methods

### Search Strategies

The Preferred Reporting Items for Systematic Reviews and Meta-analyses Protocols (PRISMA-P) checklist was used to develop the systematic review protocol (see Online Appendix) [21]. A systematic search was conducted through four databases (PubMed, Scopus, PsycINFO, and Web of Science).

The search strategies utilised Boolean operation, MeSH terms and text words related to HIV transmission, tobacco smoking and smoking cessation (Table S1). The scope of this review was restricted to peer-reviewed studies published between 2011 and 2023 in the English language and conducted on human subjects.

In this review, current smoking and smoking cessation were the primary outcomes of interest. Current smoking status was defined as participants’ self-reported current daily or intermittent tobacco smoking by the study entry. Smoking cessation was defined as self-reported quitting behaviour (e.g., ever quitting, former smoking, quitting after testing HIV-positive, and quitting in the past six months) or clinically confirmed abstinence (e.g., carbon monoxide-verified 7-day point prevalence abstinence). Secondary outcomes included intention to quit, quit attempts, adherence, uptake, and receipt of smoking cessation aids/programmes/interventions.

Our study aimed to explore associated factors of current smoking and smoking cessation rather than the effect of interventions on smoking cessation in a particular trial. Therefore, the analysis included both observational and interventional studies to comprehensively assess what could influence smoking cessation in PLWH [22, 23].

### Study Selection

Two reviewers independently reviewed and screened titles, abstracts, and full text of the selected articles in Rayyan–QCRI. For inclusion criteria, studies must: (1) be published in the English language and peer-reviewed journals; (2) empirically explore the relationship between predictors of current smoking and cessation behaviour; and (3) be conducted on PLWH. We included observational (i.e., cross-sectional and cohort studies) and experimental (i.e., randomised-controlled trials/RCTs and quasi-experimental studies) study designs. Pilot or qualitative studies, non-research articles and abstract-only papers were excluded. If the two reviewers could not reach an agreement, a third reviewer was consulted to reach a consensus. We contacted authors for non-reported estimates. Papers eligible for the systematic review were exported to Endnote X9.

### Quality Assessment

Study quality was assessed using the Cochrane risk-of-bias tool (RoB) for randomised trials and the NIH/NILBI tool for quantitative observational studies [24, 25]. For the RoB tool, grading can be ‘Low’ or ‘High’ risk of bias or can express ‘Some concerns’. Studies that fulfilled 70% of the criteria of the NILBI tool were classified as good quality.

### Data Extraction and Analysis

We extracted data from eligible studies using a standardised data extraction template (Tables S2–S4). Associated factors of the outcomes of interest were extracted for meta-analyses only if they had been assessed in at least two studies, in which at least one association was statistically significant, and if the definitions and measurements of the factors could be harmonised. Non-harmonisable factors were not meta-analysed but narratively synthesised. If studies only reported stratified analysis, each stratified analysis was considered an independent data set.

If available, we reported findings from the adjusted multivariate analyses. Odds ratios (ORs) were the effect measure of interest for the meta-analysis. Other effect measures, such as relative risks (RRs), hazard ratios (HRs), and coefficients ($\beta$), were converted to odds ratios (ORs) for consistency [26]. Non-convertible estimates, such as prevalence ratios (PR), were narratively summarised or separately meta-analysed if they met the criteria for meta-analysis. We estimated the pooled effects (pOR and pPR) separately for factors examined by different analytical methods like Poisson and logistic models, and single and multilevel models due to non-convertible measures.

The effect sizes were extracted with 95% confidence intervals (CIs). If not reported, 95%CIs were estimated based on either standard errors or p-values [27]. The pooled effect of each factor was calculated using random effect meta-analysis (due to anticipated heterogeneity) with an inverse variance weighting method that summarises effect sizes from individual studies. The weight assigned to each study was the inverse of that study’s variance. Forest plots were used to visualise the pooled effect size of each factor. We also performed the sub-analyses to compare the pooled effects between HICs and LMICs.

*I*^2^ statistics were used to quantify heterogeneity across studies [28]. An *I*^2^ value of 25–50% was classified as low, 50–75% as moderate and ≥ 75% as high heterogeneity [29]. Random-effect meta-regression was performed for factors measured in at least ten studies if moderate to high heterogeneity was suspected. Besides univariate models of meta-regression, we also built multivariate models using a stepwise removal approach. The adjusted ${R}^{2}$ reflects the proportion of between-study variance that can be explained by the model.

Meta-analysis was performed using Stata 17 SE (Stata Corp., College Station, Texas) and command *metan* [30]. We assessed publication bias using funnel plots and Egger’s test if at least ten studies were included in the meta-analysis.

## Results

### Study Selection, Characteristics, and Quality Assessment

**Fig. 1:**
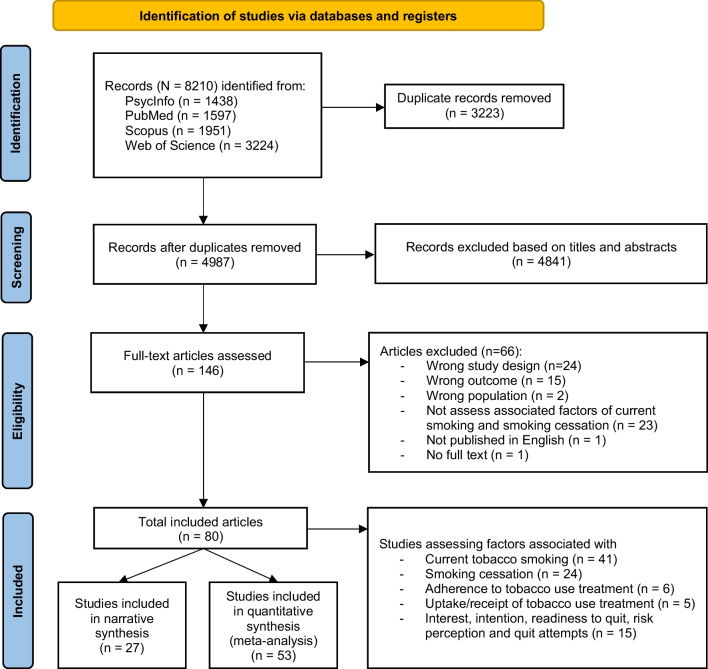
PRISMA flowchart

The search identified 8210 articles. After removing duplicates and articles based on titles, abstracts and full texts, 146 full-text articles were assessed, and 80 articles with 131,854 participants (range: 76–31,270) were included in this review [2, 19, 20, 31–107]. Of the 80 articles, 59 were conducted in HICs (51 of those in the US) and 21 in LMICs. The 80 eligible studies included cross-sectional (n = 45), cohort (n = 27), and RCT design (n = 8, all from the US). The studies explored risk factors of current smoking (n = 41), smoking abstinence (n = 24, none from LMICs) and other smoking-related outcomes (n = 26) among PLWH (some studies assessed multiple outcomes). Fifty-three of the 80 studies were included in the meta-analysis, 35 from HICs and 18 from LMICs; 38 included data on factors associated with current smoking status and 16 on those factors associated with cessation (the study by Miles et al. [66] examined both outcomes). We conducted a narrative synthesis of 27 of the total 80 studies (Fig. 1). See Table 1 for additional study characteristics.

**Table 1:** Characteristics of eligible studies by country income level

	*n*	High-income countries		Low- and middle-income countries	
		Frequency	%	Frequency	%
Total (N = 80)		59	73.75	21	26.25
Country of origin					
Australia		1	1.25		
France		1	1.25		
Germany & Austria		1	1.25		
Italy		2	2.50		
Japan		1	1.25		
Puerto Rico		1	1.25		
Switzerland		1	1.25		
USA		51	63.75		
Brazil				3	3.75
China				2	2.50
Nepal				2	2.50
Nigeria				1	1.25
Russia				1	1.25
Serbia				1	1.25
South Africa				4	5.00
Tanzania				1	1.25
Thailand				1	1.25
Uganda				2	2.50
Vietnam				2	2.50
Sub-Saharan Africa				1	1.25
With funding					
No	*1*2	8	10.00	4	5.00
Yes	*68*	51	63.75	17	21.25
Year					
2011	*4*	2	2.50	2	2.50
2012	*3*	2	2.50	1	1.25
2013	2	2	2.50	0	0.00
2014	*14*	10	12.50	4	5.00
2015	*6*	4	5.00	2	2.50
2016	*11*	10	12.50	1	1.25
2017	*6*	4	5.00	2	2.50
2018	*8*	4	5.00	4	5.00
2019	*5*	3	3.75	2	2.50
2020	*15*	13	16.25	2	2.50
2021	*4*	3	3.75	1	1.25
Study design					
Cohort	2*7*	26	32.50	1	1.25
Cross-sectional	*45*	25	31.25	20	25.00
RCT	*8*	8	10.00	0	0.00
Outcome variables of all eligible studies (N = 80)*					
Smoking abstinence	2*4*	24	30.00	0	0.00
Adherence to SCA/SCP	*6*	6	7.50	0	0.00
Current smoking	*41*	22	27.50	19	3.75
Intention to quit	*4*	3	3.75	1	1.25
Interest in quitting	2	2	2.50	0	0.00
Quit attempt	*5*	5	6.25	0	0.00
Readiness to quit	*3*	1	1.25	2	2.50
Risk perception	*1*	1	1.25	0	0.00
Uptake/receipt of SCA/SCP	*5*	5	6.25	0	0.00
Outcome variables of studies included in meta-analysis (N = 53)*					
Smoking abstinence	*16*	16	30.19	0	0.00
Current smoking	*38*	20	37.74	18	33.96

*Some studies assessed more than one outcome

All RCTs were rated as low risk of bias except for the study by Humfleet et al. which did not adjust for age differences between intervention groups [55]. Among cohort and cross-sectional studies (n = 72), two (2.8%) and twelve studies (16.7%) were graded as poor and fair quality, respectively, due to small sample sizes, self-reported data, and uncontrolled confounders, as well as attrition in cohort studies. See Tables S5 and S6 for quality grading elements.

### Findings from Quantitative Synthesis

Table 2 describes eligible studies exploring factors associated with current smoking and smoking abstinence, which were included in meta-analyses.

**Table 2:** Characteristics of studies included in meta-analyses

Short citation	Country	Outcome classifications	Study design	No of PLWH in the analysis	Measures	Analytical methods	Intervention description
Current smoking							
Akhtar-Khaleel et al. [32]	USA	*“Did you ever smoke cigarettes?” and “Do you smoke cigarettes now?”.* Participants who answered yes to both questions were categorised as current smokers	Cross-sectional	3391	PR	Poisson regression	NA
Akhtar-Khaleel et al. [33]	USA	*“Did you ever smoke cigarettes?” and “Do you smoke cigarettes now?”* Participants who answered yes to both questions were categorised as current smokers	Cohort	3357	OR	Generalized linear mixed models	NA
Amiya et al. [34]	Nepal	Current smokers are defined as self-reported smoking *“every day” or “some days” at the time of survey*	Cross-sectional	301	OR	Multivariable logistic regression	NA
Asfar et al. [106]	USA	Smoking status was assessed from two self-reported items in the data: *“Have you smoked at least 100 cigarettes in your lifetime?”* and *“Do you smoke cigarettes now?”*	Cross-sectional	152	OR	Multivariable logistic regression	NA
Batista et al. [36]	Brazil	Current smokers are identified as those who were *smokers at the time of the study or had quit smoking less than 6 months before*	Cross-sectional	Men 848 Women 497	OR	Multivariable logistic regression	NA
Bhatta et al. [38]	Nepal	Tobacco smoking is assessed using the question: *“Are you a current tobacco smoker?”*; with answer ‘yes’ or ‘no’	Cross-sectional	132	OR	Multivariable logistic regression	NA
Brath et al. [19]	Germany & Austria	*“Did you smoke at least one cigarette within the last 7 days?”*; with answer ‘yes’ or ‘no’. Smoking status was *confirmed* by exhaled carbon monoxide levels	Cross-sectional	447	OR	Multivariable logistic regression	NA
Colón-López et al. [44]	Puerto Rico	*“Have you smoked at least 100 cigarettes in your entire life?”*; with answer ‘yes’ and *“How often do you smoke cigarettes now?”*; with answer other than ‘Never’	Cross-sectional	209	OR	Multivariable logistic regression	NA
Cropsey et al. [45]	USA	Participants were classified as current smokers, ex-smokers, and non-smokers *based on responses to smoking status questions*	Cross-sectional	2874	OR	Multinomial logistic regression	NA
De Socio et al. [47]	Italy	“Current smokers” were defined as *persons who reported smoking 100 cigarettes or more during their lifetime and currently smoked every day or some days*	Cross-sectional	878	OR	Multinomial logistic regression	NA
Edwards et al. [49]	Australia	Current daily smokers included those who *self-identified as a currently smoking cigarettes and smoked on average one or more cigarettes per day*	Cross-sectional	1011	OR	Multivariable logistic regression	NA
Egbe et al. [50]	South Africa	Current tobacco use (*ever smoked cigarettes, past 30 days smoking, and having smoked* ≥ *100 sticks of cigarettes in their lifetime*)	Cross-sectional	623	RR	Poisson regression	NA
Elf et al. [51]	South Africa	Positive test results for CO and urine cotinine *tests in addition to self-report non-daily and daily smoking status*	Cross-sectional	Men 358, Women 753	OR	Multivariable logistic regression	NA
Gamarel et al. [53]	USA	Participants were asked *“Do you currently smoke cigarettes?”*	Cohort	373	OR	Multivariable logistic regression	NA
Gamarel et al. [52]	USA	Participants who reported that they *had smoked within the past 30 days* were classified as smokers	Cross-sectional	109	OR	Multivariable logistic regression	NA
Iliyasu et al. [56]	Nigeria	Regular smokers were defined as present smokers who *smoked at least one cigarette per day for one year or more*	Cross-sectional	296	OR	Multivariable logistic regression	NA
Kilibarda et al. [57]	Serbia	*“Do you currently smoke cigarettes?”*; with answer ‘yes’, ‘no’ and ‘I used to, but I do not anymore’ and the last one was recorded to no smokers	Cross-sectional	445	OR	Multivariable logistic regression	NA
Kruse et al. [59]	Uganda	Tobacco use is assessed by self-report by asking participants if they have ever used smoking tobacco/cigarettes or *chewing tobacco* and *if they have used these tobacco products in the past 30 days*	Cohort	456	PR	Poisson regression	NA
Lam et al. [60]	USA	Patients’ smoking status and use of cessation treatment were ascertained from screening and service use data in the EHR	Cross-sectional	309	PR	Poisson regression	NA
Luo et al. [63]	China	*“Have you ever smoked at least 100 cigarettes in your life?” and “Did you smoke in the past 30 days?”*; with answer ‘yes’ to both questions, then s/he was classified as a “current smoker”	Cross-sectional	455	OR	Multivariable logistic regression	NA
Mdege et al. [2]	Uganda	Participant was classified as a current smoker if they answered “daily” or “less than daily” to the question *“Do you currently smoke tobacco on a daily basis, less than daily, or not at all?”*	Cross-sectional	777	OR	Mixed-effect logistic regression	NA
Miles et al. [66]	USA	*“Have you smoked more than 20 cigarettes in your lifetime?”* and “*Do you currently smoke cigarettes?*” with answer ‘yes’ to both questions, then s/he was classified as a “current smoker”	Cohort	1413	IRR	Multivariable logistic regression	NA
Musumari et al. [68]	Thailand	Current smokers based on their questionnaire responses	Cross-sectional	364	OR	Multivariable logistic regression	NA
Mutemwa et al. [69]	South Africa	Current smokers included participants who *smoked daily or occasionally*	Cross-sectional	827	OR	Multivariable logistic regression	NA
Mwiru et al. [70]	Tanzania	Participants were asked to report on their status and there were three options, *never, yes but quit and currently smoking*	Cross-sectional	518	OR	Multivariable logistic regression	NA
Nguyen et al. [71]	Vietnam	Current smokers were those who had smoked *at least 100 cigarettes during their lifetime and had smoked in the last 30 days at the time of interview*	Cross-sectional	1133	OR	Multivariable logistic regression	NA
Ompad et al. [73]	USA	First participants were asked if they had *ever smoked cigarettes*. Those answering affirmatively were then asked if they *currently smoke cigarettes*	Cross-sectional	199	OR	Multivariable logistic regression	NA
Pacek et al. [74]	USA	Current smokers reported smoking *at least 100 cigarettes in their lifetime and within the past 30 days*	Cross-sectional	349	OR	Mixed-effect logistic regression	NA
Pacek et al. [75]	USA	Individuals reporting past 30-day smoking were current smokers	Cross-sectional	358	RR	Multinomial logistic regression	NA
Regan et al. [80]	USA	*Medical record* review for ever versus never and current versus not current smoking	Cohort	2868	RR	Generalized linear models with a log link function	NA
Reisen et al. [81]	USA	Participants were asked *if they had smoked a cigarette during the previous thirty days*	Cross-sectional	198	OR	Multivariable logistic regression	NA
Shirley et al. [86]	USA	Current smoker status was defined as having *smoked at least 100 lifetime cigarettes and an average of one cigarette daily in the last week*	Cohort	200	OR	Multivariable logistic regression	NA
Sims et al. [91]	USA	*“Do you currently smoke cigarettes?”*; with answer ‘yes’ then s/he was classified as a “current smoker”	Cross-sectional	313	OR	Binomial logistic regression	NA
Stewart et al. [94]	USA	*“Do you currently smoke cigarettes?”*; with answer ‘yes’ then s/he was classified as a “current smoker”	Cross-sectional	289	OR	Multivariable logistic regression	NA
Teixeira et al. [96]	Brazil	Current smoking was measured by the following question: *“Do you currently smoke cigarettes?”* (yes/no/ignored)	Cross-sectional	462	OR	Multivariable logistic regression	NA
Torres et al. [97]	Brazil	Current smoking was defined as answer Yes to the question *“Do you currently smoke cigarette or any other tobacco product?”*	Cross-sectional	2775	OR	Multivariable logistic regression	NA
Uthman et al. [100]	sub-Saharan Africa	Respondents were explicitly asked *“Do you currently smoke cigarettes?”* Those who responded ‘yes’ to this question were defined as current cigarette smokers	Cross-sectional	31,255	OR	Mixed-effect logistic regression	NA
Zhang et al. [104]	China	Smoking behaviour was measured by questions asking if participants *ever had smoked in the past 6 months* (yes vs. no)	Cross-sectional	2987	OR	Mixed-effect logistic regression	NA
Smoking abstinence							
Aigner et al. [31]	USA	CO-verified 24-h PPA (end of treatment-month 12) CO-verified 7-day PPA (end of treatment-month 12)	Cohort	474	Coeff	Generalized linear mixed modelling	Cell-phone intervention + NRT vs usual care + NRT
Bauer et al. [37]	USA	CO-verified 7-day PPA at 3 months	Cohort	179	OR	Single logistic regression	Varenicline + counselling vs placebo + counselling
Browning et al. [39]	USA	CO-verified 7-day PPA at 3, and 12 months	Cohort	247	OR	Multivariable logistic regression	Telephone counselling + NRT
Chew et al. [42]	USA	CO-verified 7-day PPA at 6 months	Cohort	122	OR	Multivariable logistic regression	Counselling + NRT
De Socio et al. [47]	Italy	6-month self-reported abstinence	Cohort	522	HR	Cox Proportional Hazard Regression Model	Counselling + NRT
Huber et al. [54]	Switzerland	6-month self-reported abstinence	Cohort	5805	OR	Generalized estimating equations	Counselling + NRT
Miles et al. [66]	USA	Self-reported abstinence	Cohort	1413	IRR	Generalized estimating equations	Johns Hopkins HIV clinical cohort
Moadel et al. [67]	USA	CO-verified 7-day PPA (end of treatment-3 month)	RCT	145	OR	Multivariable logistic intention-to-treat analyses	Positively smoke free counselling + NRT vs standard care + NRT
Quinn et al. [79]	USA	CO-verified 7-day PPA at 3 months	Cohort	89	OR	Multivariable logistic regression	Counselling + varenicline
Shutter et al. [89]	USA	CO-verified 7-day PPA at 3 months	Cohort	272	OR	Multivariable logistic regression	Web-based positively smoke free vs standard care
Shutter et al. [87]	USA	CO-verified 7-day PPA at 3 months	RCT	90	OR	Multivariable logistic regression	Standard care vs positively smoke free-mobile
Shutter et al. [88]	USA	CO-verified 7-day PPA (end of treatment- > 13.2 months) CO-verified 12-month PPA (end of treatment- > 13.2 months)	Cohort	194	OR	Longitudinal linear mixed effects modelling	Positively smoke free counselling
Stanton et al. [92]	USA	CO-verified 7-day PPA at 6 months	RCT	302	OR	Multivariable logistic regression	Aurora tailored counselling + NRT vs enhanced standard care + NRT
Stanton et al. [93]	USA	CO-verified 7-day PPA at 3 and 6 months	RCT	442	OR	Multivariable logistic regression	Positively smoke free (group therapy) vs standard care
Vidrine et al. [102]	USA	CO-verified 7-day PPA at 3 months	Cohort	350	Coeff	Mediation analysis	Cell phone intervention vs usual care
Zyambo et al. [105]	USA	Self-reported abstinence	Cohort	1714	HR	Cox proportional hazard regression model	Counselling

Number of studies are given in italics

*CO* carbon monoxide, *Coeff* coefficient, *HR* hazard ratio, *IRR* incidence rate ratio, *NA* not applicable, *NRT* nicotine replacement therapy, *OR* odds ratio, *PLWH* people living with HIV, *PPA* point prevalence abstinence, *RCT* randomised controlled trial, *RR* relative risk

#### Meta-analyses of Factors Associated with Tobacco Smoking and Smoking Cessation

**Fig. 2:**
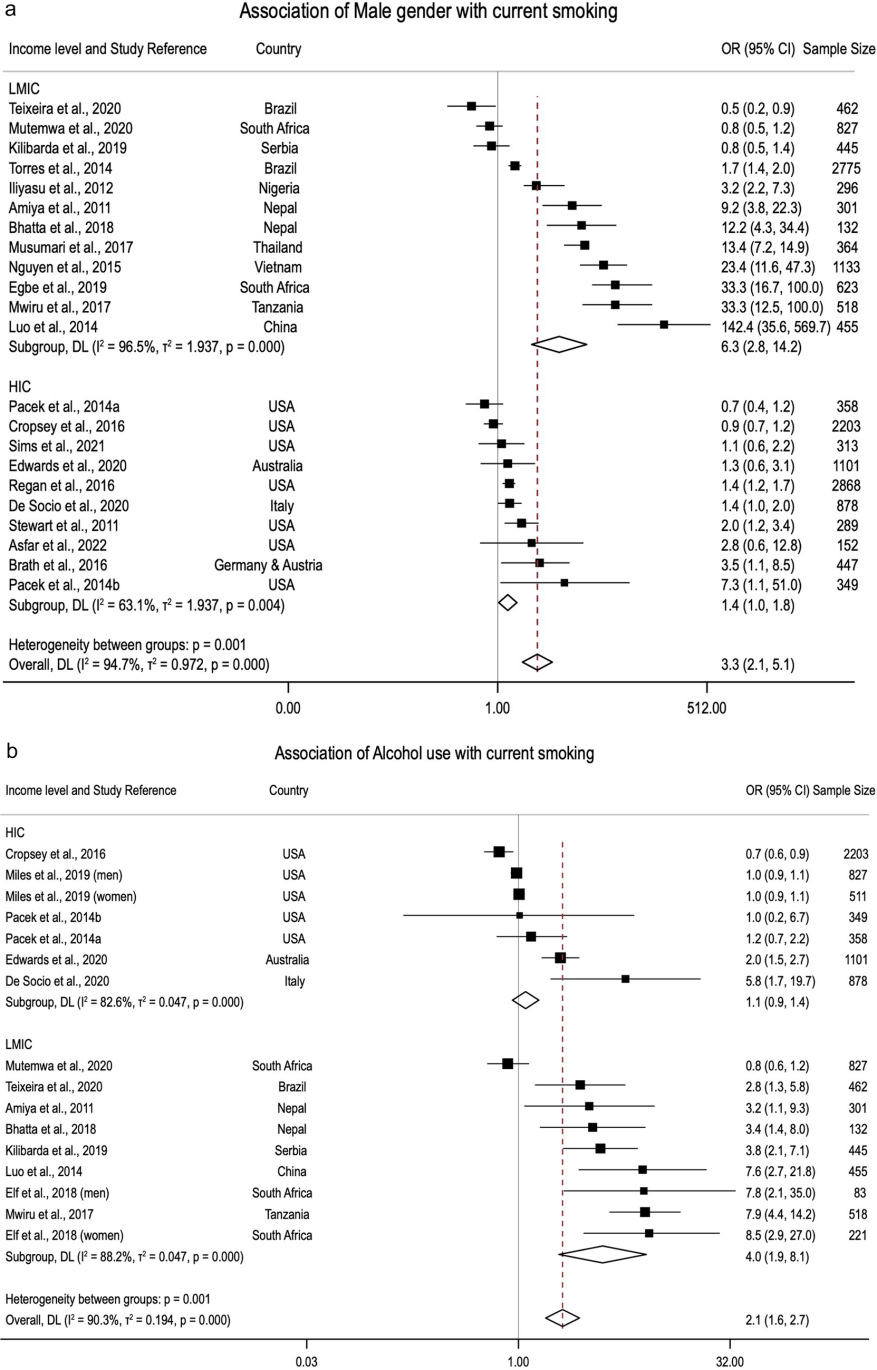

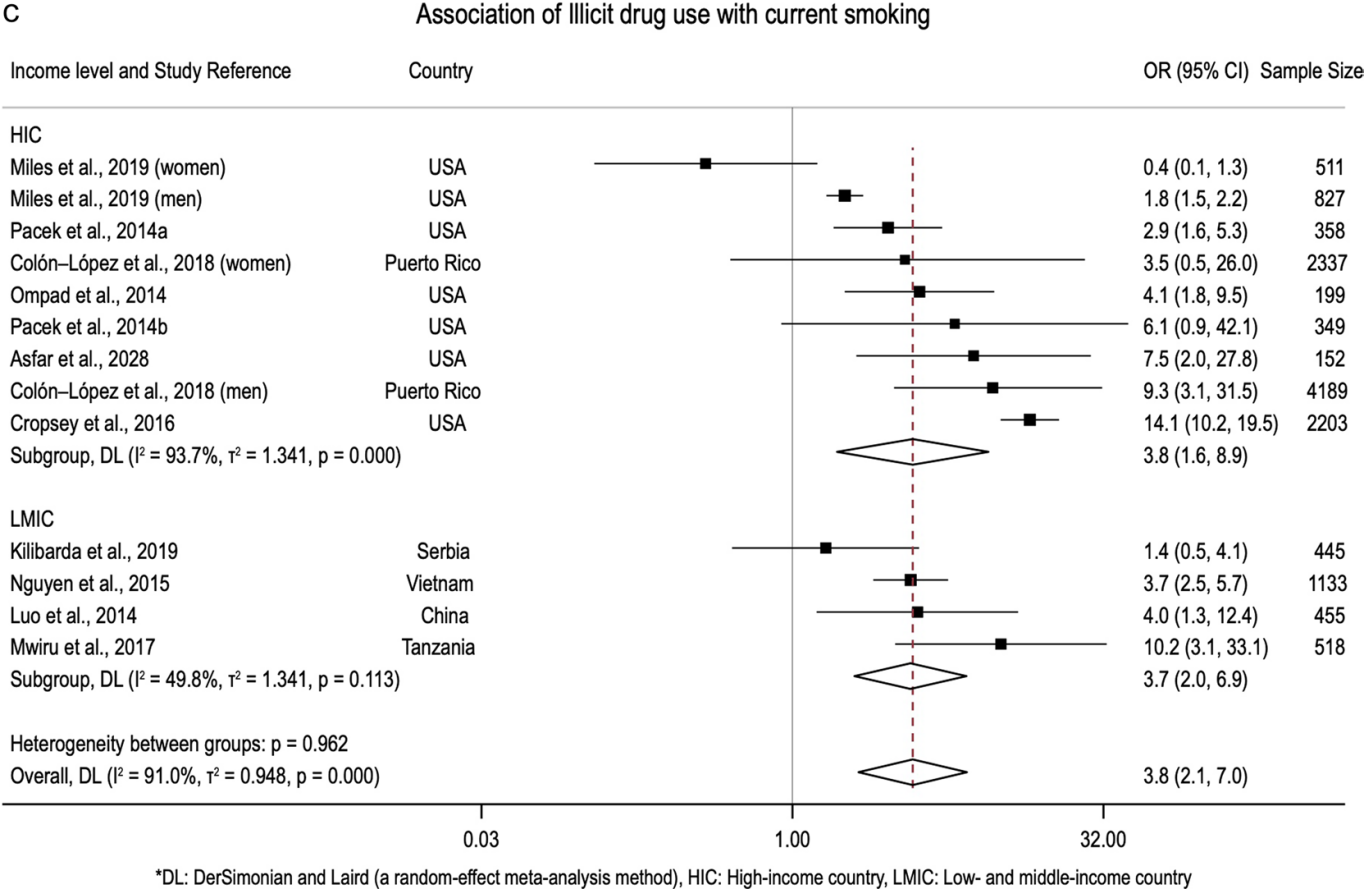
**a** Forest plot of association between current smoking and male gender. **b** Forest plot of association between current smoking and alcohol use. **c** Forest plot of association between current smoking and illicit drug use

The meta-analyses summarised 24 factors associated with current smoking and 10 associated with smoking abstinence. Operational definitions of these factors are presented in Table S7. Figure 2a–c shows the forest plots of alcohol use (n = 16), male gender (n = 22), and illicit drug use (n = 13), as these factors are eligible for heterogeneity and publication bias assessment (factors analysed by at least ten studies). See Figs. S1–S3 for the forest plots of other factors.

##### Factors Associated with Current Smoking

Men were 3.26 times more likely than women to be current smokers (n = 22; 95%CI 2.09–5.10) (Fig. 2a). This result was consistent in sub-analyses of male gender in studies from HICs (n = 10; pOR 1.35 95%CI 1.03–1.77) and LMICs (n = 12; pOR 6.26 95%CI 2.76–14.19). No tertiary education also increased the odds of current smoking (n = 5; pOR 2.11; 95%CI 1.70–2.62) (Table 3). Compared to non-Hispanic White, non-Hispanic Black ethnicity was associated with current smoking (n = 3; pOR 1.68; 95%CI 1.04–2.71). This finding was consistent in studies using the Poisson regression analytical approach (n = 2: pPR 1.09; 95%CI 1.02–1.15) (Table 3). Compared to single, divorced, or widowed PLWH, married PLWH (n = 6; pOR 0.72; 95%CI 0.55–0.95) were less likely to smoke. The reverse association was reported for those divorced or widowed versus those married or in a stable relationship (n = 3; pOR 2.14; 95%CI 1.05–4.37) (Table 3).

**Table 3:** Meta-analysis of factors associated with current smoking

Associated factor	Analytical methods	No of studies	Sample size	Pooled OR/PR* (95% CI)	${I}^{2}$
Demographic factors					
Age (years)	Logistic	13	11,014	0.99 (0.98–1.01)	73.19^a^
Age (years)	Poisson	2	3847	1.00 (1.00–1.00)	0.00
Male gender (ref: female)	Logistic	22	17,289	3.26 (2.09–5.10)	94.98^a^
Male gender (ref: female)	Poisson	2	8816	1.55 (0.64–3.78)	87.95^b^
Male gender (ref: female)	Multilevel	2	32,047	20.43 (2.26–184.94)	98.73^a^
Non-Hispanic Black (ref: non-Hispanic White)	Logistic	3	5044	1.68 (1.04–2.71)	72.97^c^
Non-Hispanic Black (ref: non-Hispanic White)	Poisson	2	11,751	1.09 (1.02–1.15)	22.43
Divorced or widowed (ref: married/in a stable relationship)	Logistic	3	1672	2.14 (1.05–4.37)	53.60
Married (ref: single, divorced, widowed)	Logistic	6	2500	0.72 (0.55–0.95)	20.35
Unemployed (ref: employed)	Logistic	8	4236	1.10 (0.82–1.47)	55.25^c^
Formal education (ref: no)	Logistic	2	433	0.76 (0.17–3.38)	83.21^c^
No tertiary education (ref: yes)	Logistic	5	6345	2.11 (1.70–2.62)	51.22
Behavioural factors					
Alcohol use (ref: no)	Logistic	16	9671	2.06 (1.56–2.72)	90.25^a^
Hazardous alcohol use (ref: no)	Logistic	6	3968	1.89 (1.33–2.69)	94.17^a^
Hazardous alcohol use (ref: no)	Poisson	3	12,207	1.41 (1.03–1.93)	87.19^a^
Binge drinking (ref: no)	Logistic	3	5327	1.64 (0.73–3.69)	91.72^a^
Alcohol & drug use (ref: no or never)	Logistic	2	511	2.55 (1.46–4.45)	0.00
Illicit drug use (ref: no or never)	Logistic	13	13,676	3.82 (2.09–6.98)	91.05^a^
Injection drug use (ref: no or never)	Logistic	2	748	5.19 (2.70–9.96)	0.00
Cocaine use (ref: no or never)	Logistic	2	3237	3.08 (2.12–4.47)	19.02
Crack use (ref: no or never)	Logistic	3	4085	5.87 (2.82–12.21)	0.00
Marijuana use (ref: no or never)	Logistic	8	3729	2.91 (1.54–5.50)	92.08^a^
Environmental factors					
Smoking living environment (ref: no)	Logistic	3	857	2.33 (0.92–5.88)	82.40^b^
Smoking partners (ref: no)	Logistic	2	820	6.78 (2.03–22.64)	75.83
Medical conditions					
COPD (ref: no)	Logistic	3	4113	1.96 (0.97–3.94)	74.22^c^
CVDs (ref: no)	Logistic	4	4560	1.32 (0.98–1.79)	0.00
Depressive symptoms (ref: no)	Logistic	6	4662	1.22 (0.96–1.55)	54.31
Depressive symptoms (continuous)	Logistic	4	993	1.05 (0.95–1.16)	70.07^c^
Tuberculosis (ref: no)	Logistic	4	4035	1.08 (0.77–1.53)	57.34
Receipt of ART (ref: no)	Logistic	7	11,698	0.92 (0.67–1.28)	39.66

*ART* anti-retroviral therapy, *COPD* Chronic obstructive pulmonary disease, *CVDs* cardiovascular diseases

*PR: Prevalence ratio (estimates for Poisson regression methods)

^a^p < 0.001, ^b^p < 0.01, ^c^p < 0.05 (p-value corresponding to Q statistics)

Alcohol use and illicit drug use were positively associated with current smoking (Fig. 2b, c). The results remained consistent with hazardous alcohol use in both logistic (n = 6, pOR 1.89; 95%CI 1.33–2.69) and Poisson models (n = 3, pPR 1.41; 95%CI 1.03–1.93) and across different types of drug use (Table 3). The effects of alcohol, hazardous alcohol, illicit drug, and marijuana use on current smoking in LMICs were larger than those in HICs (Table 4). Having smoking partners (n = 2; pOR 6.78; 95%CI 2.03–22.64) or the presence of other smokers in living and social environments (n = 3; multilevel pOR 2.33; 95%CI 0.92–5.88) was associated with current smoking (Table 3).

**Table 4:** Meta-analysis of factors associated with current smoking by country income level

Associated factor	High-income countries				Low- and middle-income countries			
	No of studies	Sample size	Pooled OR (95%CI)	${I}^{2}$	No of studies	Sample size	Pooled OR (95%CI)	${I}^{2}$
Demographic factors								
Age (years)	12	10,187	1 (0.98–1.02)	74.50^a^	1	NA		
Male gender (ref: female)	10	8958	1.35 (1.03–1.77)	63.08^b^	12	8331	6.26 (2.76–14.19)	96.51^a^
Non-Hispanic Black (ref: non-Hispanic White)	3	5044	1.68 (1.04–2.71)	72.97^c^	0			
Divorced or widowed (ref: married/in a relationship)	2	1227	5.70 (0.41–79.36)	72.39	1	NA		
Married (ref: single, divorced, widowed)	1	NA			5	2142	0.70 (0.51–0.98)	32.03
Unemployed (ref: employed)	3	1230	1.31 (0.77–2.23)	42.10	5	3006	1.02 (0.70–1.47)	62.57^a^
Formal education (ref: no)	0				2	433	0.76 (0.17–3.38)	83.21^c^
No tertiary education (ref: yes)	5	6345	2.11 (1.7–2.62)	51.22	0			
Behavioural factors								
Alcohol use (ref: no)	6	5869	1.12 (0.89–1.41)	85.26^a^	9	3444	3.97 (1.94–8.11)	88.20^a^
Hazardous alcohol use (ref: no)	3	1490	1.11 (1.04–1.18)	0.00	3	2478	3.21 (1.95–5.30)	76.40^c^
Binge drinking (ref: no)	2	2552	4.59 (0.11–197.26)	93.29^a^	1	NA		
Alcohol & drug use (ref: no or never)	2	511	2.55 (1.46–4.45)	0.00	0			
Illicit drug use (ref: no or never)	9	11,125	3.82 (1.64–8.87)	93.73^a^	4	2551	3.72 (2.00–6.95)	49.78
Cocaine use (ref: no or never)	0				2	3237	3.08 (2.12–4.47)	19.02
Crack use (ref: no or never)	0				3	4085	5.87 (2.82–12.21)	0.00
Marijuana use (ref: no or never)	3	1538	1.19 (0.63–2.24)	66.49	5	2191	5.26 (4.03–6.86)	6.42
Environmental factors								
Smoking partners (ref: no)	2	820	6.78 (2.03–22.64)	75.83^c^	0			
Smoking living environment (ref: no)	2	556	3.52 (0.99–12.52)	83.17^c^	1		NA	
Medical conditions								
COPD (ref: no)	2	1338	1.89 (0.42–8.45)^b^	87.10	1	NA		
CVDs (ref: no)	3	1785	1.00 (0.64–1.56)	0.00	1	NA		
Depressive symptoms (ref: no)	4	3899	1.18 (1.05–1.31)	0.00	2	763	1.06 (0.25–4.51)	88.79^b^
Depressive symptoms (continuous)	4	993	1.05 (0.95–1.16)	70.07^c^	0			
Tuberculosis (ref: no)	0				4	4035	1.08 (0.77–1.53)	57.34
Receipt of ART (ref: no)	5	10,942	0.99 (0.68–1.46)	48.30	2	756	0.68 (0.36–1.27)	0.00

*ART* anti-retroviral therapy, *COPD* Chronic obstructive pulmonary disease, *CVDs* cardiovascular diseases

*NA: Not applicable due to small number of study (i.e., a meta-analysis requires at least two studies)

^a^p < 0.001, ^b^p < 0.01, ^c^p < 0.05 (p-value corresponding to Q statistics)

The meta-analysis of studies in HIC studies showed a positive relationship between depressive symptoms and current smoking (n = 4; pOR 1.18; 95%CI 1.05–1.32) (Table 4). This relationship was not demonstrated in LMIC studies.

Other medical conditions, including chronic obstructive pulmonary disease (COPD) (n = 3; pOR 1.96; 95%CI 0.97–3.94), cardiovascular diseases (CVDs) (n = 4; pOR 1.32; 95%CI 0.98–1.79) and Tuberculosis (n = 4; pOR 1.08 95%CI 0.77–1.53) were positively associated with current smoking, and PLWH who received ART were less likely to smoke (n = 7; pOR 0.92; 95%CI 0.67–1.28) (Table 3). However, these associations were not statistically significant.

##### Factors Associated with Smoking Abstinence

Men were less likely to quit smoking (n = 2; pOR 0.60; 95%CI 0.37–0.98), and older age was associated with higher abstinence rates (n = 2; pOR 1.08; 95%CI 1.03–1.14) (Table 5).

**Table 5:** Meta-analysis of factors associated with smoking abstinence

Associated factor	Analytical methods	No of studies	Sample size	Pooled OR (95% CI)	${I}^{2}$
Demographic factors					
Age (years)	Logistic	2.00	447	1.08 (1.03–1.14)	0.00
Male gender (ref: female)	Multilevel	2.00	668	0.60 (0.37–0.98)	0.00
Behavioural factors					
Cocaine use (ref: no or never)	Logistic	2.00	554	0.18 (0.08–0.44)	0.00
Hazardous alcohol use (ref: no)	Logistic	4.00	8265	0.50 (0.39–0.64)	4.87
Medication adherence (ref: no)	Logistic	4.00	762	1.01 (0.98–1.04)	77.43^b^
Psychological factors					
History of depression (ref: no)	Logistic	3.00	6551	0.79 (0.68–0.93)	0.00
UCLA loneliness scores	Logistic	2.00	417	0.95 (0.91–0.99)	0.00
Smoking cessation-related factors					
FTND scale	Logistic	4.00	2006	0.82 (0.75–0.88)	0.00
Quit attempt in the past 12 months (ref: no)	Logistic	2.00	587	2.65 (1.37–5.14)	0.00
Self-efficacy scores	Logistic	2.00	440	1.60 (0.59–4.37)	78.91^c^

*FTND* Fagerström Test for Nicotine Dependence

^b^p < 0.01, ^c^p < 0.05 (p-value corresponding to Q statistics)

Those who use cocaine (n = 2; pOR 0.18; 95%CI 0.08–0–44) or displayed hazardous alcohol consumption (n = 4; pOR 0.50; 95%CI 0.39–0.64) were also less likely to abstain from smoking (Table 5). Nicotine dependence was associated with a low likelihood of abstinence rates (n = 4; pOR 0.82; 95%CI 0.75–0.88) (Table 5). However, those who had attempted to quit smoking in the last 12 months were more likely to abstain from smoking (n = 2; pOR 2.65; 95%CI 1.37–5.14) (Table 5).

People with higher UCLA loneliness scores were less likely to quit (n = 2; pOR 0.95; 95%CI 0.91–0.99) (Table 5). Besides, PLWH in HIC with a history of depression had a 21% lower likelihood of quitting smoking (n = 3; pOR 0.79; 95%CI 0.68–0.93) (Table 5). We did not find significant associations between smoking abstinence rates and medication adherence and self-efficacy.

##### Heterogeneity Assessment

We observed moderate-to-high heterogeneity (${I}^{2}$ ≥ 50%) in nine out of sixteen factors significantly associated with current smoking (Table 3). However, we assessed high heterogeneity in only three factors associated with current smoking: male gender (n = 22; ${I}^{2}$ = 94.9%; p < 0.001), alcohol use (n = 16; ${I}^{2}$ = 90.3%; p < 0.001), and illicit drug use (n = 13; 91.1%; p < 0.001) as they met the criteria for meta-regression.

Study characteristics, including geographical location (continents), ethnicity, gender, country income level, study quality, outcome definition, the proportion of current and female smokers, sample size and year, were utilised for explanatory meta-regression models (Table S8). For alcohol use, studies conducted in LMICs had 1.22 times (95%CI 0.44–1.99) higher log pOR than those in HICs, and good quality studies reduced the log pOR compared with fair quality studies ($\beta$ − 1.65; 95%CI − 3.60 to 0.29). This model explained 47.3% out of 86.2% between-study variance. Similarly, 66.4% out of 88.5% heterogeneity in effect sizes of male gender factor was explained by the proportion of current smokers and female smokers. Gender, country income level, outcome definition, and proportion of current smokers explained total between-study variations in effect sizes of illicit drug use.

##### Assessment of Small Study Effect

**Fig. 3:**
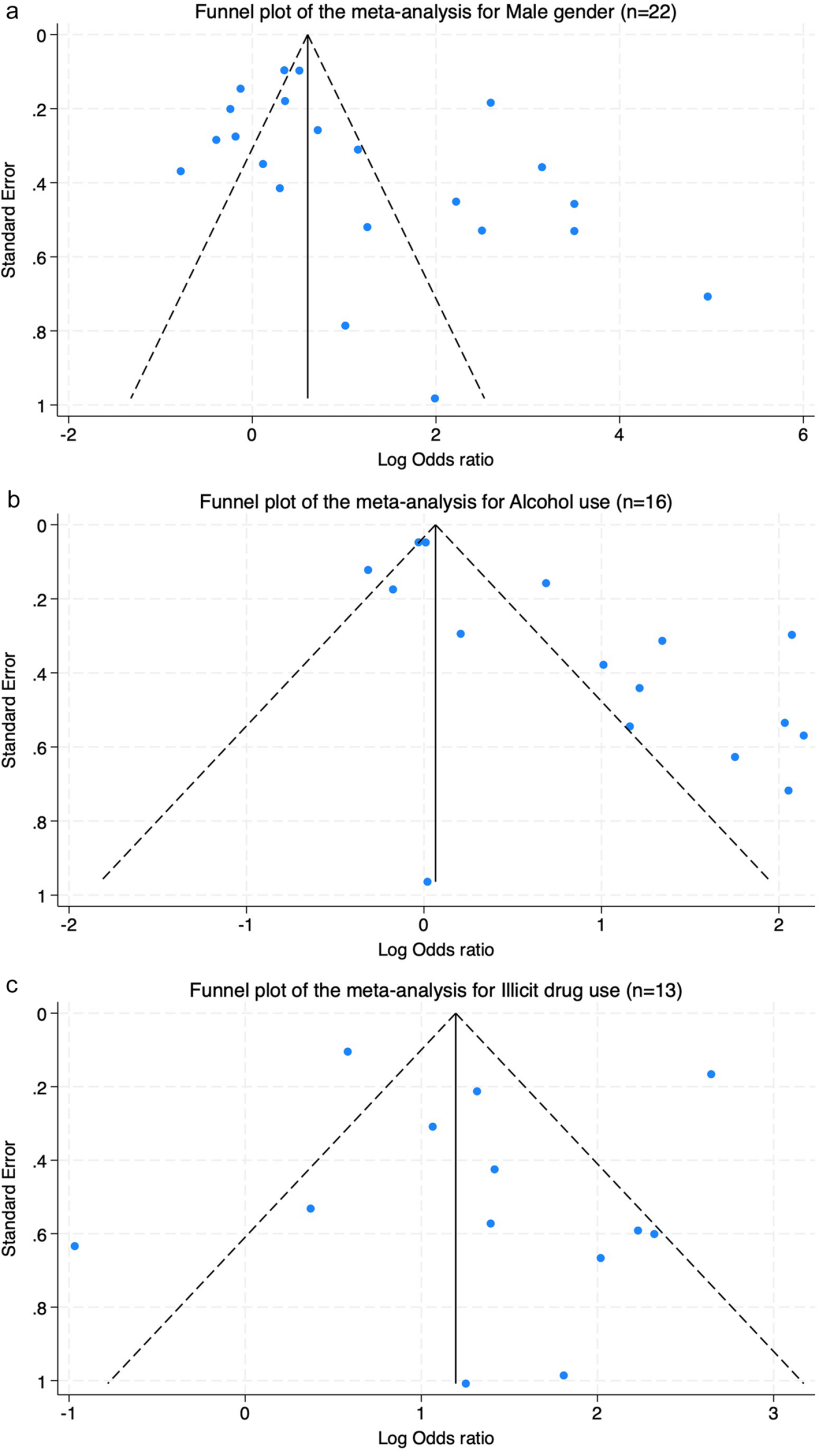
Funnel plots of the meta-analyses for **a** male gender, **b** alcohol use, and **c** illicit drug use

Funnel plots for male gender, alcohol use and illicit drug use were created to identify potential publication bias (Fig. 3a–c). Asymmetry could be subjectively seen in the plots for male gender and alcohol use. The funnel plots of illicit drug use were relatively symmetric yet lacked small studies to the left. Egger’s test confirmed the asymmetry of the funnel plot of alcohol use (0 < 0.01), which indicated small-study effects (presence of publication bias). The test did not detect small-study effects of male gender (p = 0.05) illicit drug use (p = 0.58) (Table S9).

#### Narrative Syntheses of Factors Associated with Current Smoking and Smoking Abstinence

Significant effect sizes of other factors influencing current smoking and smoking abstinence from 26 studies not eligible for meta-analysis are presented in Table S10.

##### Current Smoking

Findings from the narrative review were largely consistent with those from the meta-analyses as associations of current smoking with loneliness (living alone or homeless), substance use, and depression were frequently reported. Studies by Brath et al. and Mdege et al. found that those having a daily smoking partner (OR 8.78; 95%CI 4.49–17.17) or more than two smokers among the five closest friends (OR 3.97; 95%CI 2.08–7.59) were more likely to be current smokers [2, 19]. In addition, those of Hispanic or Latino ethnicity were less likely to smoke compared to White ethnicity. Other demographic factors, such as higher education and higher socioeconomic status, were associated with a lower likelihood of current smoking. Furthermore, low BMI, chronic diseases such as COPD and asthma, and detectable HIV viral load were associated with higher odds of current smoking.

##### Smoking Abstinence and Other Smoking Cessation-Related Outcomes

**Fig. 4:**
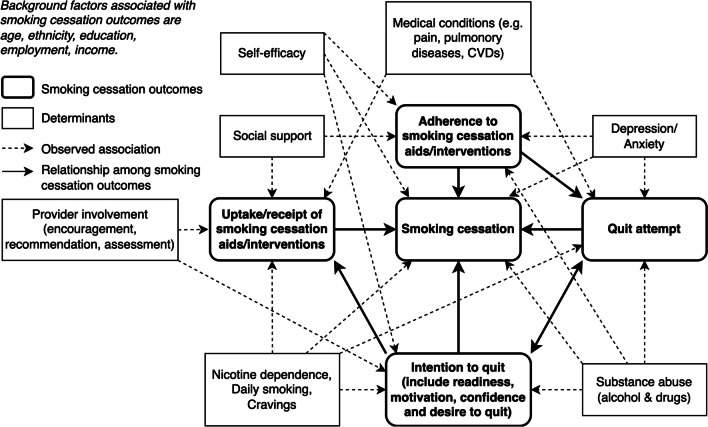
Associations of smoking cessation outcomes with multiple factors conceptualised from findings of the systematic review

Other factors significantly associated with abstinence rates and the secondary outcomes (intention to quit, quit attempt, adherence, uptake, and receipt of smoking cessation aids) were sorted into categories based on their relation and recurrence across eligible studies. These categories and their relationships were conceptually illustrated in Fig. 4. According to the model, smoking abstinence was influenced proximally by intention to quit, quit attempt, uptake, receipt, and adherence to smoking cessation aids or interventions. Distal factors, including medical conditions (e.g., pulmonary diseases, pain, and CVDs), self-efficacy, social support, depression or anxiety, nicotine dependence, substance use, and provider involvement, were indirectly associated with smoking cessation. These associations concurred with the findings from the meta-analysis.

## Discussion

### Gender Differences in Smoking

Gender differences in tobacco smoking were consistent with the Demographic and Health Survey data from 28 LMICs that reported 24.4% smoking prevalence in men and 1.3% among women living with HIV [108]. This has been explained by gender inequality that can manifest as the greater social power of men and social pressure against women smoking [109]. Indeed, the qualitative assessment by Thirlway et al. [110] revealed that smoking was widely common and socially accepted among men in Uganda. Smoking-related stigma among women could result in underreporting and create challenges in documenting the true smoking prevalence in this population [110].

### The Impact of Psychological Distress

This review found a strong association between depression and current smoking, as well as between depression and smoking abstinence. However, a systematic review has shown some inconsistency regarding the direction of this association [111]. From the qualitative studies, smoking was mainly described as a strategy for dealing with stress and depression in PLWH, which commonly resulted from several stressors, namely financial pressure, stigma, health concerns, traumatic events, and lack of social support [110, 112, 113]. Most of these stressors were identified as factors associated with smoking and unsuccessful abstinence in the descriptive synthesis, which could imply their interrelations with depression.

Furthermore, the meta-analyses found that those who were single, divorced or widowed had a higher likelihood of being current smokers, and loneliness contributed to lower abstinence rates. These results demonstrated that a lack of social support among PLWH is a risk factor for continued tobacco use.

Our review observed a positive association between adverse health conditions (e.g., CVDs, Tuberculosis and COPD) and tobacco smoking. Earlier studies found that some PLWH described worries about adverse health outcomes as their motivation to quit smoking, while others mentioned that smoking helped them feel better when they were too sick [110, 112, 113]. A qualitative study has found that life incidents and lifelong smoking habits are the primary reasons people with COPD do not quit smoking [114]. More studies, therefore, should be conducted to explore these associations further.

### Substance Use and Tobacco Smoking

This review found that alcohol, cocaine, crack, marijuana, and injection drug use significantly impacted tobacco smoking and cessation in PLWH, especially in LMICs. Among those substances, alcohol use emerged as a major determinant for current smoking in both meta-analyses and narrative syntheses. This result was in line with findings about alcohol use paired with tobacco smoking that was described as a stress-coping strategy in qualitative studies [110, 113]. Alcohol consumption was also demonstrated to increase smoking relapse through different mechanisms ranging from biochemical pathways to stress-coping theory [115, 116]. Other studies showed the other direction of the association that tobacco smoking was linked to the risk of other substance use and relapse [117, 118].

Despite the concurrence of smoking, substance use, and social and psychological challenges experienced by PLWH, their interrelationships have not been explicitly explored in the literature.

### The Role of Healthcare Providers

Substantial evidence, primarily from HICs, showed that smoking cessation interventions implemented in clinical settings delivered by healthcare providers could increase cessation rates [17]. However, our systematic review identified only four quantitative studies that described the influence of providers on disseminating knowledge and skills to quit smoking, illustrating a gap in research in healthcare settings that serve PLWH [34, 64, 77, 78]. Specifically, PLWH whose smoking status was assessed by a physician in the last 12 months were 3.34 times more likely to report readiness to quit [34]. Provider recommendations about smoking cessation also significantly increased the likelihood of interest in quitting and increased perceived risk related to smoking [77, 78]. Qualitative studies also revealed the vital role of healthcare providers in providing support, advice and treatment of tobacco use for PLWH [110, 112, 113]. This finding was consistent with two reports from Matthews et al. and Pacek et al. in high-income contexts, showing the importance of HIV care provider support regarding smoking cessation [78, 119].

Failure to screen for tobacco use, lack of training, and competing healthcare needs and priorities may create barriers to engaging PLWH in treatment [120]. Unfortunately, most providers in LMICs have limited access to training resources to deliver tobacco use treatment for PLWH [112, 121]. PLWH’s regular contact with the healthcare system presents an important opportunity to intervene. Thus, provider training for tobacco use treatment among PLWH is greatly needed in LMICs.

### Strengths and Limitations

To our knowledge, this systematic review is the first to apply descriptive and quantitative methods to synthesise evidence about factors influencing smoking and cessation behaviour among PLWH. Findings from our different approaches provided a more comprehensive understanding of predictors of tobacco smoking and cessation behaviour in this understudied population. The review revealed the lack of RCTs of smoking cessation intervention for PLWH in LMICs.

Several drawbacks of the study need to be discussed. Eligible studies have measured smoking abstinence differently, either based on self-reporting or biochemical verification of tobacco smoking. Even though self-reported data have been shown to be accurate, the potential bias cannot be fully ignored [122]. Similarly, biochemical confirmation of smoking abstinence increases the rigour and validity of cigarette smoking and abstinence measurements. However, this measure is not practical to measure long-term abstinence due to costs and implementation challenges [123]. Hence, the results should be interpreted in the context of this limitation. This study did not consider levels of tobacco smoking, such as heavy or light smoking since all included studies mainly reported current smoking as a binary variable. Similarly, pooling reported effect size estimates was challenging due to different time points of abstinence rate assessment. The intention to use the follow-up time as an explanatory factor of potential heterogeneity was not fulfilled due to the small number of studies assessing factors associated with smoking abstinence.

We attempted to harmonise independent variables such as age, education, substance use, and depression from eligible studies based on definitions and measurement scales to make them plausible for the meta-analysis. This process was rigorously conducted to minimise the risk of selection bias and inaccuracy. The poor precision of certain pooled effect sizes, such as smoking partners and crack use, could be due to either the small number of studies or the wide variation in the effect sizes of individual studies.

Finally, heterogeneity of some significant determinants of current smoking remained unexplained due to the few studies. The small number of studies or imprecision of effect sizes may also lead to false low heterogeneity; therefore, the findings should be interpreted in the broader context of existing research.

### Conclusion

Smoking is more prevalent in PLWH, who are less likely to quit than the general population. Although studies have explored tobacco smoking and smoking cessation behaviour among the PLWH population, there is a lack of particular reviews that include both HICs and LMICs and a full range of study designs to guide the development and implementation of effective treatments.

This review provided a comprehensive summary of multiple factors associated with smoking and cessation in PLWH, which have implications for future intervention design. Particularly, interventions for PLWH need to be tailored to sociocultural and gender differences and should integrate with screening and treatment for mental health and substance use that addresses these risk factors to optimise cessation outcomes. Given the essential role of HIV care providers, professional training that enables them to effectively assess and assist patients in smoking cessation should be offered. Lastly, RCTs should be conducted to examine the effectiveness of smoking cessation aids/interventions for PLWH in LMICs where the need is greater. Successful implementation of such interventions would reduce the burden of HIV/AIDS and HIV-related comorbidities and increase treatment outcomes in PLWH.

### Supplementary Information

Below is the link to the electronic supplementary material.

Supplementary file1 (DOCX 4235 KB)

## Data Availability

Not applicable.

## Code Availability

Stata 17 SE (Stata Corp., College Station, Texas).
