# Supplementary material for: Factors Influencing Tobacco Smoking and Cessation Among People Living with HIV: A Systematic Review and Meta-analysis
Source: AIDS Behav. 2024 Mar 13;28(6):1858–81. doi: 10.1007/s10461-024-04279-1 (PMC11161546; doi:10.1007/s10461-024-04279-1)

Appendix

Thanh Hoang

SCHOOL OF PUBLIC HEALTH AND COMMUNITY MEDICINE – SAHLGRENSKA ACADEMY

Tables & Figures

**TABLE OF CONTENT**

**Tables**

[Table S1 Search strategy 1](#_Toc125717803)

[Table S2 Data extraction of studies assessed current smoking (not eligible to be included in meta-analysis) 2](#_Toc125717804)

[Table S3 Data extraction of studies assessed smoking abstinence (not eligible to be included in meta-analysis) 3](#_Toc125717805)

[Table S4 Data extraction of studies assessed other secondary outcomes 5](#_Toc125717806)

[Table S5 Quality assessment of cohort and cross-sectional studies 10](#_Toc125717807)

[Table S6 Quality assessment of randomised controlled trials 14](#_Toc125717808)

[Table S7 Operational definitions of associated factors with current smoking and smoking abstinence included in meta-analyses 15](#_Toc125717809)

[Table S8 Meta-regression of effects of alcohol use, male gender and illicit drug use on current smoking 17](#_Toc125717810)

[Table S9 Publication bias – Egger's tests 19](#_Toc125717811)

[Table S10 Narrative synthesis of factors influencing current smoking, smoking abstinence and other secondary outcomes 20](#_Toc125717812)

**Figures**

[Figure S1 Forest plots of factors influencing current smoking by country income level (studies using Logistic regression) 31](#_Toc127874710)

[Figure S2 Forest plots of factors influencing current smoking (studies using Multilevel & Poisson regression) 36](#_Toc127874711)

[Figure S3 Forest plots of factors influencing smoking abstinence 38](#_Toc127874712)

Table S1 Search strategy

| PICO/PECO | Description | Search | Block 1 – HIV transmission & Tobacco smoking |
| --- | --- | --- | --- |
| Population | Smokers living with HIV/AIDS | #1  #2  #3 | HIV [MeSH] OR acquired immunodeficiency syndrome [MeSH] OR HIV [Title/Abstract] OR human immunodeficiency virus [Title/Abstract] OR AIDS[Title/Abstract] OR acquired immune deficiency syndrome [Title/Abstract]  Smok* [Title/Abstract] OR tobacco use* [Title/Abstract] OR tobacco use [MeSH] OR smoker [MeSH] OR smoking [MeSH] OR nicotine  #1 AND #2 |
| Interventions/  Exposures |  |  | **N/A** |
| Comparators |  |  | **N/A** |
|  |  |  | **Block 2 – Smoking cessation behaviour** |
| Outcomes | A process of quitting or stopping tobacco smoking.  *Primary outcome:*   - Smoking abstinence   *Secondary outcome:*   - Current/continued smoking - Quit attempts - Intention to quit - Readiness to quit - Adherence to/uptake of SCA/SCI | #4 | Smoking cessation [MeSH] OR tobacco use cessation [MeSH] OR smoking cessation [Title/Abstract] OR quit* OR abstinen* OR stop* OR cess* or cease* OR giv* up OR intention* OR readiness* |
|  |  |  | **Search hits** |
|  |  | #5 | #3 AND #4 |
|  |  |  | **Limits** |
|  |  | #6 | Limit #5 to English language and Human species using filters |

*SCA: Smoking cessation aid, SCI: Smoking cessation intervention

Table S2 Data extraction of studies assessed current smoking (not eligible to be included in meta-analysis)

| Short citation | Country of Origin | Outcome definition | Study design | No of PLWH in the analysis | Measures | Analytical methods |
| --- | --- | --- | --- | --- | --- | --- |
| de Dios et al., 2016 | USA | Any **self-reported tobacco use in the past 7 days (even one puff) at the 6-month follow-up verified using a 24-hour biochemical measure** of smoking via carbon monoxide exhalation testing with less than 10 parts per million (ppm) was considered non-smoking. | Cohort | 318 | Coefficient | Standardised probit regression coefficient |
| Lasser et al., 2018 | Russia | The primary outcome was **heavy smoking** (among regular smokers), defined as smoking > 20 cigarettes per day. | Cross-sectional | 1123 | Odds ratio | Multivariable logistic regression |
| Mdodo et al., 2015 | USA | Current cigarette smokers were defined as persons who reported that they **had smoked 100 cigarettes or more during their lifetime and currently smoked every day or some days.** | Cross-sectional | 3981 | Prevalence Difference (percentage points) | Multivariable logistic regression |

Table S3 Data extraction of studies assessed smoking abstinence (not eligible to be included in meta-analysis)

| Short citation | Country of Origin | Study design | Intervention description | Outcome definition | Outcome classifications | Length of follow-up | No of PLWH in the analysis | Measures | Analytical methods |
| --- | --- | --- | --- | --- | --- | --- | --- | --- | --- |
| Ashare et al., 2019 | USA | RCT | Varenicline + Counselling vs Placebo + Counselling | Smoking cessation **primary outcomes** were **7-day** point-prevalence abstinence **at Weeks 12 and 24**, based on no **self-reported** tobacco use (not even a puff) during the 7 days preceding the assessment and a **CO ≤8ppm**.   Secondary outcomes included **7-day** point-prevalence abstinenc**e at Week 18**, defined as no self-reported tobacco use for the duration of the timeframe (based on previous varenicline trials) | CO-verified 7-day PPA at 3 and 6 months | Weeks 12, 18 and 24 ≈ at 3, 4.5 and 6 months | Week 12: 150, Week 24: 141 | Odds ratio | Multivariable logistic regression |
| Buchberg et al., 2016 | USA | Cohort | Cell Phone Intervention vs Usual Care | The primary outcome was **7-day** smoking abstinence at a **3-month follow-up**, which was **biochemically verified** with expired CO level. | CO-verified 7-day PPA at 3 months | 3,6 and 12 months | 474 | Odds ratio | Multivariable logistic regression |
| Humfleet et al., 2013 | USA | RCT | Individual Counselling, Computer-based intervention, and NRT only | Participants were coded as abstinent only if they (self-)**reported** no cigarette use, not even a puff, in the last **7 days** and if their **CO ≤10 ppm**. | CO-verified 7-day PPA (end of treatment-M13) | 12, 24, 36, and 52 weeks | 169 | Odds ratio | Multivariable logistic regression |
| Kim et al., 2020 | USA | Cohort | Telephone-based, video-call, cessation counselling (HIV-tailored) vs Telephone-based, voice-call (attention-control) counselling | Smoking abstinence was assessed using the **7-day**, point-prevalence abstinence **at a 3-month follow-up** that could be **verified** with a salivary cotinine test. Abstinence was defined as having not smoked a single puff during the past 7 days. | CO-verified 7-day PPA at 3 months | 1, 2 and 3 months | 69 | Odds ratio | Multivariable logistic regression |
| Taniguchi et al., 2020 | Japan | Cohort | Japanese Smoking Cessation Therapy (Counselling + Varenicline) | Success in quitting smoking at the last session of SCT was defined as the condition that subjects replied that they **quit smoking for at least the previous 2 weeks**, which was **verified by the CO ≤7 ppm** concentration in expired air. Those who dropped out of the SCT from the second to the fifth session were treated as not having succeeded in quitting smoking. | CO-verified 2-week PPA | 12 weeks | 77 | Odds ratio | Multivariable logistic regression |
| Triant et al., 2020 | USA | Cohort | Counselling + NRT vs Usual care | Smoking status was assessed **at 6-month follow-up** by **self-report** of the number of days in the **last 30** that participants smoked and the average number of cigarettes per day smoked on those days. Those reporting no days of smoking were classified as quit; among those reporting continued smoking, smoking intensity (defined as the number of cigarettes per day × days smoked/30) was calculated. | 30-day self-reported abstinence at 6 months | 6 months | 172 HIV+ | Odds ratio | Multivariable logistic regression |
| Tseng et al., 2017 | USA | RCT | Standard care + Varenicline vs  Text message (TM) support + Varenicline vs  TM support + Varenicline + Telephone-based adherence-focused Counselling | **Self-reported** **7-day** point prevalence smoking abstinence was verified by a carbon monoxide (**CO <8 ppm**) and measured at 1, 4, 8, and 12 weeks. Participants with missing data due to loss-to-follow-up or withdrawal/discharge from the study were considered non-abstinent (intent-to-treat approach). | CO-verified 7-day PPA (end of treatment-3M) | 1, 4, 8, and 12 weeks | 158 | Odds ratio | Generalised linear mixed-effect models |
| Vidrine et al., 2018 | USA | Cohort | No intervention, but patients provided smoking cessation information | Participants were considered abstinent if they **self-reported** not smoking (not even a single puff) in the past **7 days** and had an expired CO <7ppm | CO-verified 7-day PPA at 3, 6, 9 and 12 months | At 3, 6, 9, and 12 months | 378 | Odds ratio, Mean difference | Multivariable linear regression, Multivariable logistic regression |

Table S4 Data extraction of studies assessed other secondary outcomes

| Short citation | Country | Study design | Intervention description | Outcome definition | Number of PLWH included in the analysis | | Measures | | Analytical methods | | |
| --- | --- | --- | --- | --- | --- | --- | --- | --- | --- | --- | --- |
| ADHERENCE | | | | | | | | | | | |
| Browning et al., 2016 | USA | Cohort | Telephone counselling + NRT | Adherence to the pharmacotherapy was defined as the proportion of weeks that pharmacotherapy was taken as prescribed out of 12 weeks. Patient adherence to the tobacco dependence treatment protocol was measured by self-reported use of pharmacotherapy collected by the nurse during the weekly calls (range 1–12 weeks), calculated as a dichotomous variable ≥80 versus <80% of 12 weeks of drug taken as prescribed. | 247 | | Odds ratio | | Multivariable logistic regression | | |
| de Dios et al., 2016 | USA | Cohort | (1) Standard Care counselling + NRT (SC) vs (2) motivationally enhanced treatment + NRT (motivational enhancement) | Nicotine patch adherence was measured using retrospective self-reports of NRT patches collected at each follow-up visit. Participants were prompted to recall the number of days they used the dispensed NRT patches during the corresponding follow-up interval. This variable was further collapsed into a nine-item scale, which corresponds to the 8 weeks of NRT patches dispensed (0 = no patches used; 1= 7 days of patch use; 2 = 8–14 days; 3 = 15–21 days; 4 = 22–28 days; 5 = 29–35 days; 6 = 36–42 days; 7 = 43–49 days; 8 = 50–56; and 9 = greater than 56 days of patches use). | 318 | | Coefficient | | Standardised probit regression coefficient | | |
| Quinn et al., 2020 | USA | Cohort | Varenicline | Varenicline adherence was assessed using the timeline follow-back method and blister-pack collection as done previously. We assessed the total number of pills taken out of the total pills prescribed and computed an overall proportion of medication adherence (adherence defined by taking ≥80% of prescribed medication). If a discrepancy regarding the number of pills taken arose between blister-packs and what was reported during timeline follow-back, the amount in the blister-pack was recorded and used. | 89 | | Odds ratio | | Multivariable logistic regression | | |
| Shelley et al., 2015 | USA | Cross-sectional | NA | Adherence was defined as taking at least 80% of prescribed Varenicline in the previous 4 weeks (i.e., at the 1-month follow-up visit), as determined by pill count. Participants who did not bring their medication bottles for pill count were considered non-adherent. | 127 | | Coefficient | | Weighted least squares estimator (WLSMV) | | |
| Shutter et al., 2014 | USA | RCT | Web-based Positive Smoke-Free vs Standard care | Visited all webpages, More interactive clicks, More time spent logged into the site | 136 | | Odds ratio | | Multivariable logistic regression | | |
| Tseng et al., 2017 | USA | RCT | Standard care + Varenicline vs  Text message (TM) support + Varenicline vs  TM support + Varenicline + Telephone-based adherence-focused Counselling | Adherence was defined as taking ≥80 % of prescribed Varenicline since the last visit, as determined by pill count and was assessed at 1, 4, 8 and 12-week follow-up visits. Participants who did not bring their medication bottles for pill count or who did not come back for follow-up visits were considered non-adherent (intent-to-treat approach). | 158 | | Odds ratio | | Generalised linear mixed-effect models | | |
| UPTAKE & RECEIPT OF SMOKING CESSATION AIDS | | | | | | | | | | | |
| Bui et al., 2020 | USA | Cohort | Ask-Advise-Connect + NRT | Number of counselling calls completed and receipt of NRT. | | 214 | | Odds ratios | | Multivariable logistic regression |  |
| Lam et al., 2020 | USA | Cross-sectional | NA | Patients' smoking status and use of cessation treatment were ascertained from screening and service use data in the EHR. Data on the use of cessation pharmacotherapy were obtained from KPNC pharmacy databases. Patients were considered to have used cessation treatment if they: 1) filled a cessation-related prescription medication (e.g., nicotine replacement therapy, bupropion or Varenicline), 2) attended a group cessation class, or 3) completed an individual cessation coaching session. | | 1123 | | Prevalence ratio | | Multivariable Poisson regression |  |
| McQueen et al., 2014 | USA | Cross-sectional | NA | Use of NRT products, cessation medication use, participation in Counselling, and consent to genetic testing to determine the best smoking cessation treatment in the next 6 months were assessed using 5 single-item measures developed for this study based on previous work. Response options ranged from 1 = not at all willing to 5 = extremely willing. | | 146 | | Odds ratios | | Univariable logistic regression |  |
| Pacek et al., 2014c | USA | Cross-sectional | NA | Dichotomous variables were created based on Supporters' responses to questions regarding lifetime NRT/medication use. | | 267 | | Odds ratios | | Multivariable logistic regression |  |
| Shahrir et al., 2016 | USA | Cohort | NA | The primary outcome of interest was a dichotomous measure identifying receipt of any smoking cessation pharmacotherapy (NRT, bupropion, and Varenicline) based on outpatient prescriptions filled from VHA pharmacy data in the CDW 0–365 days following the date of the completed survey. | | 814 | | Risk ratios (RR/ARR) | | Marginal multivariable log-linear regression models with an over-dispersed Poisson working model |  |
| INTENTION TO QUIT | | | | | | | | | | |  |
| Cioe et al., 2017 | USA | Cross-sectional | NA | For quit intentions, a three-category variable was created: (1) immediate (i.e., within the next 30 days); (2) future (i.e., within 6 months or more); and (3) no plans to quit. | | 91 | | Odds ratios | | Multinomial logistic regression |  |
| LaRowe et al., 2020 | USA | Cross-sectional | NA | A single item was used to assess intention (yes/no) to quit smoking within the next six months ("Are you seriously considering quitting smoking within the next six months?"). This item was previously used to assess intention to quit in national surveys. | | 76 | | Odds ratios, Coefficient (Beta) | | Mixed-effect logistic regression |  |
| Shapiro et al., 2011 | South Africa | Cross-sectional | NA | Respondents classified intent to quit as 1) seriously considering stopping in <30 days; 2) seriously considering stopping in <12 months; 3) wanting to stop but has no plan; or 4) not wanting to quit. Intent to quit was defined as high (response 1 or 2) or low (3 or 4). | | 150 | | Odds ratios | | Multivariable logistic regression |  |
| Vidrine et al., 2018 | USA | Cohort | NA | Intention to quit smoking was assessed with a single Likert-type scale in which participants were asked the question "Do you plan to quit smoking?". Responses ranged from 1 (lowest intention to quit) to 7 (highest intention to quit). | | 378 | | Odds ratios, Mean difference | | Multivariable linear regression, Multivariable logistic regression |  |
| INTEREST IN QUITTING | | | | | | | | | | |  |
| Pacek et al., 2014 | USA | Cross-sectional | NA | Dichotomous variables were created based on Supporters' responses to questions regarding interest in quitting and lifetime NRT/medication use. | | 267 | | Odds ratios | | Multivariable logistic regression |  |
| Pacek et al., 2017 | USA | Cross-sectional | NA | Participants reported current interest in quitting smoking (yes/no). | | 275 | | Odds ratios | | Multivariable logistic regression |  |
| READINESS TO QUIT | | | | | | | | | | |  |
| Amiya et al., 2011 | Nepal | Cross-sectional | NA | Participants not ready to quit are those reporting (1) no intention to quit at all or (2) no intention to quit within the next 6 months or (3) those reporting an intention to quit in the next 1-6 months. Participants ready to quit are those reporting an intention to quit within the next 30 days and reporting a previous quit attempt in the past 12 months | | 301 (current tobacco smoking outcome)  131 (readiness to quit outcome) | | Odds ratios | | Multivariable logistic regression |  |
| Donnelly et al., 2021 | USA | Cross-sectional | NA | The Readiness Ruler (RR) was used as a continuous measure of readiness to quit cigarette smoking. Participants indicated their readiness to quit cigarette smoking within the next month on a scale of 1 to 10 with 1 = "not at all ready to quit smoking within the next 30 days" and 10 = "actively quitting smoking within the next 30 days" [36]. Participants also answered a binary question about whether they were ready to quit smoking in the next 30 days (yes/no). | | NA | | Odds ratios, Coefficient (Beta) | | Multivariable logistic regression, Generalised additive model (GAM) |  |
| Nguyen et al., 2015 | Vietnam | Cross-sectional | NA | Motivation to quit smoking, based on the Contemplation Ladder, is a common measure of readiness to quit smoking. The question "Currently, what is your thinking about quitting smoking?" was used to measure motivation to quit. Responses included "No thought of quitting" was classified as having no motivation to quit, "Think I should quit but not quite ready", "Starting to think about how to change my smoking patterns", and "Take action to quit" were categorised as having motivation to quit. | | 409 | | Odds ratios | | Multivariable logistic regression |  |
| QUIT ATTEMPT | | | | | | | | | | |  |
| Barré et al., 2021 | France | Cohort | NA | Tobacco smoking quit attempt was defined as the time between baseline and the first transition from 'current smoker' to 'former smoker' status | | 695 | | Hazard ratios | | Cox Proportional Hazard Regression Model |  |
| Browning et al., 2016 | USA | Cohort | NA | The number of 24-h quit attempts in the past year is a secondary measure. | | 247 | | Odds ratios | | Multivariable logistic regression |  |
| De Socio et al., 2020 | Italy | Cohort | NA | Primary outcome was patient's self-reported abstinence for at least 6 months. | | 522 | | Hazard ratios | | Cox Proportional Hazard Regression Model |  |
| Shahrir et al., 2020 | USA | Cross-sectional | NA | Participants responded to the question "During the past 12 months, have you stopped [smoking] for more than one day because you were trying to quit?". Only participants who responded "Yes" or "No" were included in the analysis (n = 1721; 975 HIV-infected, 746 HIV-uninfected) | | 975 PLWH, 746 non-PLWH | | Odds ratios | | Multivariable logistic regression |  |
| Vijayaraghavan et al., 2014 | USA | Cohort | NA | We defined a quit attempt as a self-report of no cigarette consumption in the past 30 days at a study follow-up visit (i.e., point prevalence abstinence lasting for 30 days) | | 210 | | Odds ratios | | Random-effects multivariable logistic regression |  |
| RISK PERCEPTION | | | | | | | | | | |  |
| Pacek et al., 2018 | USA | Cross-sectional | NA | "Indicate how much you believe [smokers/non-smokers] are at risk for developing the following health problems on a scale from 1 to 10." And "If you did not smoke cigarettes, indicate what you believe your risk is for developing the following health problems on a scale from 1 to 10." Responses ranged from 1 to 10, with 1 anchored at "very low risk" and 10 anchored at "very high risk". | | 276 | | Coefficient (Beta) | | Multivariable linear regression |  |

Table S5 Quality assessment of cohort and cross-sectional studies

| Short citations | Q1 | Q2 | Q3 | Q4 | Q5 | Q6 | Q7 | Q8 | Q9 | Q10 | Q11 | Q12 | Q13 | Q14 | Grading | Comments |  |
| --- | --- | --- | --- | --- | --- | --- | --- | --- | --- | --- | --- | --- | --- | --- | --- | --- | --- |
| Bui et al., 2020 | Y | Y | Y | N | N | Y | Y | Y | Y | N | Y | Y | N | N | **Poor** | Many lost to follow-ups. Authors could not examine differences by individual characteristics, including other substance use, sexual identities, and sexual orientations. |  |
| Taniguchi et al., 2020 | Y | Y | Y | Y | Y | Y | Y | NA | Y | Y | Y | Y | N | N | **Poor** | 33.6% drop out, not controlled for SES and social support, a small sample (n=77 at baseline) |  |
| Bauer et al., 2021 | Y | Y | Y | Y | N | Y | Y | N | Y | Y | Y | Y | NA | Y | **Fair** | Self-reported Varenicline adherence |  |
| Chew et al., 2014 | Y | Y | Y | Y | N | Y | Y | N | N | N | Y | Y | Y | Y | **Fair** | The study lacked a comparison measure, as abstinence among non-SCP participants was unknown. Small sample size. No biochemical confirmation. |  |
| De dios et al., 2016 | Y | Y | Y | Y | N | Y | Y | N | Y | N | Y | Y | N | Y | **Fair** | Self-reported adherence to NRT (recall bias), 27% lost to follow-ups |  |
| De socio et al., 2020 | Y | Y | Y | Y | N | Y | Y | N | Y | Y | N | Y | Y | Y | **Fair** | No clinically confirmed smoking abstinence (self-reported) |  |
| Donnelly et al., 2021 | Y | Y | Y | Y | N | N | NA | NA | Y | N | Y | Y | NA | Y | **Fair** | Small sample size, self-reported data |  |
| Gamarel et al., 2016 | Y | Y | Y | Y | N | Y | Y | N | Y | N | Y | Y | N | Y | **Fair** | Not generalisable due to specific population (MSM), over 20% lost to follow-ups |  |
| Huber et al., 2012 | Y | Y | Y | Y | N | Y | Y | Y | Y | Y | N | Y | Y | Y | **Fair** | No clinical confirmation of smoking cessation |  |
| Kim et al., 2020 | Y | Y | Y | Y | N | Y | Y | NA | Y | Y | Y | Y | NI | Y | **Fair** | Small sample size (<100 both arms) |  |
| Shutter et al., 2020 | Y | Y | Y | Y | Y | Y | Y | NA | Y | Y | Y | Y | N | Y | **Fair** | 40% lost to follow-up, but treat missing outcome data as non-abstinent (worst scenario) |  |
| Triant et al., 2020 | Y | Y | Y | Y | NA | Y | Y | NA | Y | N | N | Y | Y | Y | **Fair** | Our primary outcome was self-reported smoking cessation and was not biochemically validated. Small sample size |  |
| Vidrine et al., 2018 | Y | Y | Y | Y | N | Y | Y | Y | Y | Y | Y | Y | N | Y | **Fair** | Despite a robust method to handling missing data, it remains possible that our observed effects could be biased, due to potential non-ignorable missingness (>20%). |  |
| Vijayaraghavan et al., 2014 | Y | Y | Y | Y | N | Y | Y | N | Y | Y | Y | Y | N | Y | **Fair** | Self-reported data, 30% loss to follow-ups |  |
| Aigner et al., 2017 | Y | Y | Y | Y | N | Y | Y | N | Y | Y | Y | Y | Y | Y | **Good** |  |  |
| Akhtar-khaleel et al., 2016 | Y | Y | Y | Y | N | Y | Y | N | Y | Y | Y | Y | NI | Y | **Good** |  |  |
| Akhtar-khaleel et al., 2017 | Y | Y | Y | Y | N | N | NA | NA | Y | NA | Y | Y | NA | Y | **Good** |  |  |
| Amiya et al., 2011 | Y | Y | Y | Y | N | N | NA | NA | Y | NA | Y | Y | NA | Y | **Good** |  |  |
| Asfar et al., 2021 | Y | Y | Y | Y | N | N | NA | NA | Y | NA | Y | Y | NA | Y | **Good** |  |  |
| Batista et al., 2014 | Y | Y | Y | Y | N | N | NA | NA | Y | NA | Y | Y | NA | Y | **Good** |  |  |
| Barré et al., 2021 | Y | Y | Y | Y | N | N | NA | NA | Y | NA | Y | Y | NA | Y | **Good** |  |  |
| Bhatta et al., 2018 | Y | Y | Y | Y | Y | N | NA | NA | Y | NA | Y | Y | NA | Y | **Good** | Has sample size calculation and random sampling |  |
| Brath et al., 2016 | Y | Y | Y | Y | N | N | NA | NA | Y | NA | Y | Y | NA | Y | **Good** | Male dominant population |  |
| Browning et al., 2016 | Y | Y | Y | Y | N | Y | Y | Y | Y | Y | Y | Y | Y | Y | **Good** |  |  |
| Buchberg et al., 2016 | Y | Y | Y | Y | N | Y | Y | N | Y | Y | Y | Y | N | Y | **Good** | Missing data were believed to be random. |  |
| Cioe et al., 2017 | Y | Y | Y | Y | N | N | NA | NA | Y | N | Y | Y | NA | Y | **Good** | Convenience sampling |  |
| Colón-lópez et al., 2018 | Y | Y | Y | Y | N | N | NA | NA | Y | NA | NA | Y | NA | Y | **Good** |  |  |
| Cropsey et al., 2016 | Y | Y | Y | Y | N | N | NA | NA | NA | NA | Y | Y |  | Y | **Good** | Convenience sampling |  |
| De socio et al., 2020 | Y | Y | Y | Y | N | N | NA | NA | Y | NA | Y | Y | NA | Y | **Good** | Self-reported data |  |
| Edwards et al., 2020 | Y | Y | Y | Y | N | N | NA | NA | Y | NA | Y | Y | NA | Y | **Good** | Convenience sample |  |
| Egbe et al., 2019 | Y | Y | Y | Y | N | N | NA | NA | Y | NA | Y | Y | NA | Y | **Good** |  |  |
| Elf et al., 2018 | Y | Y | Y | Y | N | N | NA | NA | Y | NA | Y | Y | NA | Y | **Good** |  |  |
| Gamarel et al., 2020 | Y | Y | Y | Y | N | N | NA | NA | Y | N | Y | N | NA | Y | **Good** |  |  |
| Iliyasu et al., 2012 | Y | Y | Y | Y | Y | N | NA | NA | Y | NA | Y | Y | NA | Y | **Good** |  |  |
| Kilibarda et al., 2019 | Y | Y | Y | Y | N | N | NA | NA | Y | NA | Y | Y | NA | Y | **Good** | Non-probability sampling |  |
| Kruse et al., 2014 | Y | Y | Y | Y | N | Y | Y | N | Y | N | Y | Y | Y | Y | **Good** | Self-reported smoking cessation |  |
| Lam et al., 2020 | Y | Y | Y | N | N | N | NA | NA | Y | NA | Y | Y | NA | Y | **Good** |  |  |
| Larowe et al., 2020 | Y | Y | Y | Y | N | N | NA | NA | Y | N | Y | Y | NA | Y | **Good** |  |  |
| Lasser et al., 2018 | Y | Y | Y | Y | N | N | NA | NA | Y | NA | Y | Y | NA | Y | **Good** |  |  |
| Luo et al., 2014 | Y | Y | Y | Y | Y | N | NA | NA | Y | NA | Y | Y | NA | Y | **Good** | Self-reported data |  |
| Mcqueen et al., 2014 | Y | Y | Y | Y | N | N | NA | NA | NA | NA | Y | NA | NA | Y | **Good** | Convenience sampling |  |
| Mdege et al., 2021 | Y | Y | Y | Y | Y | N | NA | NA | Y | NA | Y | Y | NA | Y | **Good** |  |  |
| Mdodo et al., 2015 | Y | Y | Y | N | N | NA | NA | NA | Y | NA | Y | Y | NA | Y | **Good** | Even though subjects (PLWH and non-PLWH) were recruited from different settings, they were assessed separately; therefore this did not affect the quality. |  |
| Miles et al., 2019 | Y | Y | Y | Y | N | Y | Y | N | Y | N | Y | Y | Y | Y | **Good** |  |  |
| Musumari et al., 2017 | Y | Y | Y | Y | N | N | NA | NA | Y | NA | Y | Y | NA | Y | **Good** | No random sampling. |  |
| Mutemwa et al., 2020 | Y | Y | Y | Y | N | NA | NA | NA | Y | NA | Y | Y | NA | Y | **Good** |  |  |
| Mwiru et al., 2017 | Y | Y | Y | Y | N | N | NA | NA | Y | NA | Y | Y | NA | Y | **Good** |  |  |
| Nguyen et al., 2014 | Y | Y | Y | Y | N | N | NA | NA | Y | NA | Y | Y | NA | Y | **Good** | Self-reported data |  |
| Nguyen et al., 2015 | Y | Y | Y | Y | N | N | NA | NA | Y | NA | Y | Y | NA | Y | **Good** | Convenience sampling, and recall bias due to self-reported data. |  |
| Ompad et al., 2014 | Y | Y | Y | Y | N | N | NA | NA | Y | NA | Y | Y | NA | Y | **Good** | Self-reported data |  |
| Pacek et al., 2014 | Y | Y | Y | N | N | N | NA | NA | NA | NA | Y | Y | NA | Y | **Good** |  |  |
| Pacek et al., 2014 | Y | Y | Y | N | N | N | NA | NA | Y | NA | Y | Y | NA | Y | **Good** |  |  |
| Pacek et al., 2014 | Y | Y | Y | Y | N | N | NA | NA | NA | NA | Y | Y | NA | Y | **Good** | self-reported data |  |
| Pacek et al., 2018 | Y | Y | Y | Y | N | N | NA | NA | Y | NA | Y | Y | NA | Y | **Good** | Self-reported data |  |
| Pacek et al., 2017 | Y | Y | Y | Y | N | N | NA | NA | Y | NA | Y | Y | NA | Y | **Good** |  |  |
| Parienti et al., 2017 | Y | Y | Y | Y | N | Y | Y | NA | Y | NA | Y | Y | Y | Y | **Good** |  |  |
| Quinn et al., 2020 | Y | Y | Y | Y | N | Y | Y | Y | Y | Y | Y | Y | Y | Y | **Good** |  |  |
| Real et al., 2021 | Y | Y | Y | Y | N | NA | NA | NA | Y | NA | Y | Y | NA | Y | **Good** |  |  |
| Regan et al., 2016 | Y | Y | Y | N | N | Y | Y | NA | Y | NA | Y | Y | Y | Y | **Good** |  |  |
| Reisen et al., 2011 | Y | Y | Y | Y | N | N | NA | NA | Y | NA | Y | Y | NA | Y | **Good** |  |  |
| Shahrir et al., 2016 | Y | Y | Y | N | N | NA | NA | NA | Y | NA | Y | Y | NA | Y | **Good** | Even though subjects (PLWH and non-PLWH) were recruited from different settings, they were assessed separately; therefore, this did not affect the quality. |  |
| Shahrir et al., 2020 | Y | Y | Y | N | N | Y | Y | N | Y | N | Y | Y | Y | Y | **Good** |  |  |
| Shapiro et al., 2011 | Y | Y | Y | Y | N | N | NA | NA | NA | NA | Y | Y | NA | Y | **Good** | Small sample size |  |
| Shelley et al., 2015 | Y | Y | Y | Y | N | Y | Y | Y | Y | NA | Y | Y | Y | Y | **Good** |  |  |
| Shirley et al., 2013 | Y | Y | Y | Y | N | N | Y | NA | NA | NA | Y | Y | Y | Y | **Good** |  |  |
| Shutter et al., 2014 | Y | Y | Y | Y | N | Y | NA | N | Y | Y | Y | Y | NI | Y | **Good** |  |  |
| Sims et al., 2021 | Y | Y | Y | Y | N | N | NA | NA | NA | NA | Y | Y | NA | Y | **Good** |  |  |
| Stewart et al., 2011 | Y | Y | Y | Y | N | N | NA | NA | Y | NA | Y | Y | NA | Y | **Good** |  |  |
| Teixeira et al., 2020 | Y | Y | Y | Y | Y | N | NA | NA | Y | NA | Y | Y | NA | Y | **Good** |  |  |
| Torres et al., 2014 | Y | Y | Y | Y | N | NA | NA | NA | NA | NA | Y | Y | NA | Y | **Good** | Measures were self-reported, and no biochemical verification was made; individuals were prone to memory bias, especially the former smokers, and social desirability bias. |  |
| Uthman et al., 2016 | Y | Y | Y | Y | N | NA | NA | NA | NA | NA | Y | Y | NA | Y | **Good** |  |  |
| Vidrine et al., 2015 | Y | Y | Y | Y | N | Y | Y | N | Y | N | Y | Y | NI | Y | **Good** | The sample's demographic profile (e.g., predominately minority, low-income male) may limit the generalisability of the findings |  |
| Zhang et al., 2018 | Y | Y | Y | Y | N | NA | NA | NA | Y | N | Y | Y | NA | Y | **Good** | Data collection was based on self-report and was subjected to potential recall and misclassification biases for key variables. |  |
| Zyambo et al., 2019 | Y | Y | Y | Y | N | Y | Y | Y | Y | N | Y | Y | NI | Y | **Good** | Self-reported data |  |
|  | | | | | | | | | | | | | | | | | |
| 1. Was the research question or objective in this paper clearly stated? | | | | | | | | | | | | | | | | | |
| 2. Was the study population clearly specified and defined? | | | | | | | | | | | | | | | | | |
| 3. Was the participation rate of eligible persons at least 50%? | | | | | | | | | | | | | | | | | |
| 4. Were all the subjects selected or recruited from the same or similar populations (including the same time period)? Were inclusion and exclusion criteria for being in the study prespecified and applied uniformly to all participants? | | | | | | | | | | | | | | | | | |
| 5. Was a sample size justification, power description, or variance and effect estimates provided? | | | | | | | | | | | | | | | | | |
| 6. For the analyses in this paper, were the exposure(s) of interest measured prior to the outcome(s) being measured? | | | | | | | | | | | | | | | | | |
| 7. Was the timeframe sufficient so that one could reasonably expect to see an association between exposure and outcome if it existed? | | | | | | | | | | | | | | | | | |
| 8. For exposures that can vary in amount or level, did the study examine different levels of the exposure as related to the outcome (e.g., categories of exposure, or exposure measured as continuous variable)? | | | | | | | | | | | | | | | | | |
| 9. Were the exposure measures (independent variables) clearly defined, valid, reliable, and implemented consistently across all study participants? | | | | | | | | | | | | | | | | | |
| 10. Was the exposure(s) assessed more than once over time? | | | | | | | | | | | | | | | | | |
| 11. Were the outcome measures (dependent variables) clearly defined, valid, reliable, and implemented consistently across all study participants? | | | | | | | | | | | | | | | | | |
| 12. Were the outcome assessors blinded to the exposure status of participants? | | | | | | | | | | | | | | | | | |
| 13. Was loss to follow-up after baseline 20% or less? | | | | | | | | | | | | | | | | | |
| 14. Were key potential confounding variables measured and adjusted statistically for their impact on the relationship between exposure(s) and outcome(s)? | | | | | | | | | | | | | | | | | |

Table S6 Quality assessment of randomised controlled trials

| **Intention-to-treat** | **Study ID** | **Experimental** | **Comparator** | **Outcome** | **Weight** | **D1** | **D2** | **D3** | **D4** | **D5** | **Overall** |  |  |
| --- | --- | --- | --- | --- | --- | --- | --- | --- | --- | --- | --- | --- | --- |
|  | Ashare et al, 2019 | Varenicline | Placebo | Abstinence rate (AR) | 1 |  |  |  |  |  |  |  | Low risk |
|  | Humfleet et al, 2013 | Computer-based internet + NRT & SH (NRT only) | Individual Counselling | AR | 1 |  |  |  |  |  |  |  | Some concerns |
|  | Moadel et al, 2012 | Positive Smoke Free (PSF) program | Standard care (SC) | AR | 1 |  |  |  |  |  |  |  |  |
|  | Shuter et al, 2018 | PSF-M programme | SC | AR | 1 |  |  |  |  |  |  | D1 | Randomisation process |
|  | Shuter et al, 2014 | PSF-W | SC | Adherence | 1 |  |  |  |  |  |  | D2 | Deviations from the intended interventions |
|  | Stanton et al, 2015 | Aurora project | Enhanced-SC | AR | 1 |  |  |  |  |  |  | D3 | Missing outcome data |
|  | Stanton et al, 2020 | PSF programme | SC | AR | 1 |  |  |  |  |  |  | D4 | Measurement of the outcome |
|  | Tseng et al, 2017 | SC+Text message (TM) & SC+TM+ABT | SC | AR, Adherence | 1 |  |  |  |  |  |  | D5 | Selection of the reported result |

Table S7 Operational definitions of associated factors with current smoking and smoking abstinence included in meta-analyses

| Associated factors | Definition |
| --- | --- |
| Age | Continuous variable with the unit of years |
| Alcohol & drug use | Ever or current past month use (No or Never as reference) |
| Alcohol use | Self-reported current or past month use (No as reference) |
| Male gender | Female gender as reference |
| Binge drinking | Binary variable defined by AUDIT-C ≥4 (<4 as reference) |
| Cocaine use | Ever or current past month use (No or Never as reference) |
| COPD | Chronic obstructive pulmonary disease (No as reference) |
| Crack use | Ever or current past month use (No or Never as reference) |
| CVDs | Cardiovascular diseases (No as reference) |
| Depressive symptoms | Binary variable defined by cut-off points (different scales e.g., PHQ, CES-D ect.) indicating moderate-severe depressive symptoms |
| Depressive symptoms (continuous) | CES-D or PHQ-9 with higher scores indicating more depressive symptoms |
| Divorced or widowed | In a relationship as reference |
| Formal education | No as reference |
| FTND scale | Continuous variable - Higher Fagerström Test for Nicotine Dependence scores indicate higher nicotine dependence |
| Hazardous alcohol use | Binary variable defined by AUDIT-C ≥3 (<3 as reference) |
| History of depression | No as reference |
| Illicit drug use | Ever or current past month use (No or Never as reference) |
| Injection drug use | Ever or current past month use (No or Never as reference) |
| Latino ethnicity | Ethnicity with non-Latino as reference |
| Loneliness | Continuous variable measured by UCLA Scale with higher scores indicating more loneliness |
| Marijuana use | Ever or current past month use (No or Never as reference) |
| Medication adherence | Adherence to pharmacotherapy for smoking cessation (No as reference) |
| Married | Single/Unmarried as reference |
| No tertiary education | Yes as reference |
| Non-daily smokers | Daily smokers as reference |
| Non-Hispanic Black | Ethnicity with non-Hispanic White as reference |
| Quit attempt in the past 12 months | No as reference |
| Receipt of ART | No as reference |
| Self-efficacy | Continuous variable measured by different scales with higher scores indicating more self-efficacy |
| Smoking living environment | Having ≥1 smokers among family members and closest friends (No as reference) |
| Smoking partners | No as reference |
| Tuberculosis | No as reference |
| Unemployed | Employed as reference |

Table S8 Meta-regression of effects of alcohol use, male gender and illicit drug use on current smoking

|  | Alcohol use | | Male gender | | Illicit drug use | |
| --- | --- | --- | --- | --- | --- | --- |
|  | **Univariable model** | **Multivariable model** | **Univariable model** | **Multivariable model** | **Univariable model** | **Multivariable model** |
|  | **Coeff (95%CI)** | **Coeff (95%CI)** | **Coeff (95%CI)** | **Coeff (95%CI)** | **Coeff (95%CI)** | **Coeff (95%CI)** |
| Continent Africa (ref) | |  |  |  |  |  |
| Asia | 0.13 (-1.24, 1.50) |  | 1.15 (-0.43, 2.72) |  | -0.97 (-3.85, 1.91) |  |
| Australia | -0.64 (-2.36, 1.08) |  | -1.56 (-4.17, 1.06) |  |  |  |
| Europe | 0.17 (-1.35, 1.70) |  | -1.42 (-3.18, 0.34) |  | -1.95 (-5.32, 1.42) |  |
| North America | -1.36 (-2.49, -0.24)* |  | -1.47 (-2.93, -0.00) |  | -0.98 (-3.53, 1.57) |  |
| South America | -0.32 (-2.20, 1.57) |  | -1.95 (-3.91, 0.01) |  |  |  |
| $\boldsymbol{I}^{\boldsymbol{2}}$residual | **84.49%** |  | **88.61%** |  | **92.95%** |  |
| Adjusted $\boldsymbol{R}^{\boldsymbol{2}}$ | **36.54%** |  | **52.23%** |  | **-17.41%** |  |
| Ethnicity African/African American (ref) | | |  |  |  |  |
| Asian | 0.1 (-1.36, 1.55) |  | 1.4 (0.02, 2.78) |  | -0.97 (-3.79, 1.84) |  |
| Mixed | -0.89 (-2.00, 0.21) |  | -1.33 (-2.48, -0.18)* |  | -1.08 (-3.55, 1.40) |  |
| $\boldsymbol{I}^{\boldsymbol{2}}$residual | **88.86%** |  | **86.53%** |  | **92.30%** |  |
| Adjusted $\boldsymbol{R}^{\boldsymbol{2}}$ | **16.91%** |  | **59.67%** |  | **-14.73%** |  |
| Gender Both (ref) |  |  |  |  |  |  |
| Men | -0.14 (-1.76, 1.49) |  |  |  | -0.34 (-1.59, 0.90) | -1.74 (-2.15, -1.32)*** |
| Women | 0.00 (-1.58, 1.59) |  |  |  | -1.75 (-3.57, 0.06) | -3.04 (-4.36, -1.72)** |
| $\boldsymbol{I}^{\boldsymbol{2}}$residual | **90.27%** |  |  |  | **82.99%** |  |
| Adjusted $\boldsymbol{R}^{\boldsymbol{2}}$ | **-17.71%** |  |  |  | **24.58%** |  |
| Income level HIC (ref) | |  |  |  |  |  |
| LMIC | 1.08 (0.28, 1.88)* | 1.22 (0.44, 1.99)** | 1.34 (0.09, 2.59)* |  | -0.01 (-1.34, 1.32) | -1.84 (-2.49, -1.19)*** |
| $\boldsymbol{I}^{\boldsymbol{2}}$residual | **86.31%** |  | **94.12%** |  | **91.76%** |  |
| Adjusted $\boldsymbol{R}^{\boldsymbol{2}}$ | **39.02%** |  | **17.94%** |  | **-12.62%** |  |
| Quality grading Fair (ref) | |  |  |  |  |  |
| Good | -0.94 (-3.20, 1.33) | -1.65 (-3.60, 0.29) | 0.93 (-2.29, 4.14) |  |  |  |
| $\boldsymbol{I}^{\boldsymbol{2}}$residual | **90.45%** |  | **94.96%** |  |  |  |
| Adjusted $\boldsymbol{R}^{\boldsymbol{2}}$ | **-0.14%** |  | **-3.97%** |  |  |  |
| Current smoking defined by Biochemical confirmed (ref) | | |  |  |  |  |
| Electronic health record | -0.07 (-2.47, 2.33) |  | 1.17 (-2.87, 5.22) |  |  |  |
| Self-reported | -1.47 (-3.00, 0.06) |  | -0.14 (-3.53, 3.26) |  | -0.06 (-2.43, 2.31) | 1.62 (0.08, 3.17)* |
| $\boldsymbol{I}^{\boldsymbol{2}}$residual | **89.08%** |  | **95.16%** |  | **91.79%** |  |
| Adjusted $\boldsymbol{R}^{\boldsymbol{2}}$ | **24.92%** |  | **-5.98%** |  | **-11.33%** |  |
| % Current smokers | -0.01 (-0.04, 0.02) |  | -0.02 (-0.06, 0.02) | 0.05 (0.01, 0.09)* | -0.02 (-0.05, 0.01) | -0.05 (-0.07, -0.03)** |
| $\boldsymbol{I}^{\boldsymbol{2}}$residual | **89.35%** |  | **94.05%** |  | **90.74%** |  |
| Adjusted $\boldsymbol{R}^{\boldsymbol{2}}$ | **-1.07%** |  | **0.61%** |  | **5.55%** |  |
| % Current female smokers | -0.02 (-0.04, 0.01) |  | -0.05 (-0.07, -0.02)*** | -0.08 (-0.12, -0.05)*** | -0.01 (-0.03, 0.01) |  |
| $\boldsymbol{I}^{\boldsymbol{2}}$residual | **90.89%** |  | **91.42%** |  | **89.66%** |  |
| Adjusted $\boldsymbol{R}^{\boldsymbol{2}}$ | **6.56%** |  | **48.62%** |  | **-10.20%** |  |
| Sample Size | 0 (-0.00, -0.00)* |  | 0 (-0.00, 0.00) |  | 0 (-0.00, 0.00) |  |
| $\boldsymbol{I}^{\boldsymbol{2}}$residual | **89.98%** |  | **94.37%** |  | **82.50%** |  |
| Adjusted $\boldsymbol{R}^{\boldsymbol{2}}$ | **26.55%** |  | **1.62%** |  | **21.91%** |  |
| Year | -0.02 (-0.22, 0.17) |  | -0.13 (-0.34, 0.09) |  | -0.1 (-0.36, 0.16) |  |
| $\boldsymbol{I}^{\boldsymbol{2}}$residual | **90.86%** |  | **94.87%** |  | **87.61%** |  |
| Adjusted $\boldsymbol{R}^{\boldsymbol{2}}$ | **-6.77%** |  | **1.92%** |  | **-1.71%** |  |
| $\boldsymbol{I}^{\boldsymbol{2}}$residual (Multivariable model) |  | **86.23%** |  | **88.52%** |  | **0%** |
| Adjusted $\boldsymbol{R}^{\boldsymbol{2}}$ (Multivariable model) |  | **47.29%** |  | **66.43%** |  | **100%** |
| N | 16 | 16 | 22 | 22 | 13 | 13 |

**: p<0.05; **: p< 0.01; ***: P<0.001*

Table S9 Publication bias – Egger's tests

|  | Number of studies | Test of H0: no small-study effects |
| --- | --- | --- |
| Male gender | 22 | 0.052 |
| Alcohol use | 16 | 0.001 |
| Illicit drug use | 13 | 0.581 |

Table S10 Narrative synthesis of factors influencing current smoking, smoking abstinence and other secondary outcomes

| Study Reference | Associated factors of current smoking | Measure Est. (95% CI) |
| --- | --- | --- |
|  | **Age** |  |
| Batista et al., 2014 (men) | Age ≥40 (vs <40) | OR 1.56 (1.11–2.20) |
| Lam et al., 2020 | Age (10-year interval) | PR 0.80 (0.77–0.84) |
| Mdodo et al., 2015 | Age 40-49 (vs 18-29) | PD 10.80 (4.70–16.80) |
| Pacek et al., 2014b | Age 35+ (vs 18-34) | OR 0.14 (0.03–0.79) |
| Torres et al., 2014 | Age 31-40 (vs <30) | OR 0.69 (0.52–0.90) |
| Uthman et al., 2016 | Age 25-34 (vs 18-24) | OR 1.95 (1.65–2.22) |
| Uthman et al., 2016 | Age 35-44 (vs 18-24) | OR 1.64 (1.37–1.90) |
| Uthman et al., 2016 | Age 45+ (vs 18-24) | OR 1.30 (1.06–1.51) |
|  | **Ethnicity** |  |
| Akhtar-Khaleel et al., 2016b | Non-Hispanic Black (vs non-Hispanic White – among light smokers) | OR 1.44 (1.16–1.73) |
| Akhtar-Khaleel et al., 2016b | Non-Hispanic Black (vs non-Hispanic White – among heavy smokers) | OR 1.22 (1.03–1.42) |
| Cropsey et al., 2016 | Hispanic ethnicity (vs Other than White and African American) | OR 0.55 (0.37–0.81) |
| De Socio et al., 2020 | non-White (vs White) | OR 0.28 (0.17–0.47) |
| Gamarel et al., 2016 | Latino ethnicity (vs No) | OR 0.20 (0.07–0.58) |
| Mdodo et al., 2015 | non-Hispanic Black (vs Hispanic/Latino) | PD 7.00 (1.90–12.1) |
| Mdodo et al., 2015 | non-Hispanic White (vs Hispanic/Latino) | PD 10.90 (5.90–15.9) |
| Mdodo et al., 2015 | Other ethnicity than non-Hispanic Black and White (vs Hispanic/Latino) | PD 12.00 (3.20–20.9) |
| Regan et al., 2016 | White (vs Others) | OR 1.28 (1.08–1.54) |
|  | **Education** |  |
| Akhtar-Khaleel et al., 2016a | High school diploma or less (vs Graduate work or more) | PR 1.23 (1.19–1.27) |
| Akhtar-Khaleel et al., 2016a | Some college or college degree (vs Graduate work or more) | PR 1.10 (1.07–1.13) |
| Batista et al., 2014 (women) | Having no literacy (vs Yes) | OR 2.01 (1.17–3.45) |
| Brath et al., 2016 | Tertiary education level (vs Primary) | OR 0.43 (0.15–0.79) |
| Iliyasu et al., 2012 | Secondary education level (vs No formal education) | OR 1.52 (1.01–6.25) |
| Iliyasu et al., 2012 | Tertiary education level (vs No formal education) | OR 2.63 (1.08–6.67) |
| Kruse et al., 2014 | Not complete primary school (vs Yes) | PR 2.65 (1.43–4.91) |
| Mdodo et al., 2015 | High school education (vs More than high school) | PD 9.50 (5.70–13.4) |
| Mdodo et al., 2015 | Less than high school education (vs More than high school) | PD 13.6 (9.00–18.1) |
| Teixeira et al., 2020 | Schooling ≤ 9 years (vs ≥ 13 years) | OR 2.35 (1.09–5.08) |
| Torres et al., 2014 | Schooling ≥ 9 years (vs < 9 years) | OR 0.69 (0.57–0.84) |
| Uthman et al., 2016 | Low neighbourhood literacy rate (vs High) | OR 1.28 (1.14–1.42) |
| Uthman et al., 2016 | Primary education (vs Secondary or higher) | OR 1.38 (1.24–1.53) |
|  | **Socioeconomic position** |  |
| Akhtar-Khaleel et al., 2016a | Unemployed (vs No) | PR 1.11 (1.08–1.13) |
| Batista et al., 2014 (men) | Monthly income < 2 minimum wage (vs ≥2) | OR 2.13 (1.42–3.19) |
| Bhata et al., 2018 | Formal employee (vs Unemployed) | OR 3.95 (1.36–11.47) |
| Edwards et al., 2020 | Social security–benefits–pension as income resource (vs Salary) | OR 1.61 (1.12–2.31) |
| Gamarel et al., 2016 | Low income (vs No) | OR 3.90 (1.87–8.13) |
| Kruse et al., 2014 | Manual jobs (vs Unemployed) | PR 3.07 (1.04–9.04) |
| Lam et al., 2020 | Lower neighbourhood income (vs No) | PR 1.13 (1.00–1.27) |
| Musumari et al., 2017 | Sufficient family finance (vs Insufficient) | OR 0.36 (0.15–0.84) |
| Mdodo et al., 2015 | Income below the poverty level (vs At or above) | PD 8.30 (3.90–12.60) |
| Uthman et al., 2016 | Low intensity of deprivation (vs High) | OR 1.53 (1.08–1.96) |
| Uthman et al., 2016 | Low neighbourhood poverty rate (vs High) | OR 1.25 (1.09–1.43) |
| Uthman et al., 2016 | Middle SES (vs Richer) | OR 1.29 (1.12–1.46) |
| Uthman et al., 2016 | Poorer SES (vs Richer) | OR 1.62 (1.38–1.90) |
| Uthman et al., 2016 | Low neighbourhood unemployment rate (vs High) | OR 1.11 (1.01–11.43) |
| Uthman et al., 2016 | Unemployed (vs No) | OR 0.79 (0.68–0.90) |
|  | **Relationship & living condition** |  |
| Batista et al., 2014 (women) | Living alone or in shelters (vs Living with family) | OR 2.23 (1.17–4.22) |
| Elf et al., 2018 (women) | Number of people in living situation | OR 0.90 (0.80–0.98) |
| Mdodo et al., 2015 | Ever incarcerated (vs No) | PD 8.80 (1.80–15.8) |
| Mdodo et al., 2015 | Homeless (vs No) | PD 11.90 (6.10–17.8) |
|  | **BMI** |  |
| Lam et al., 2020 | Obese BMI >30 (vs Normal BMI 18.5-25) | PR 0.64 (0.56–0.73) |
| Lam et al., 2020 | Overweight BMI 25-30 (vs Normal BMI 18.5-25) | PR 0.77 (0.69–0.85) |
| Lam et al., 2020 | Underweight BMI <18.5 (vs Normal BMI 18.5-25) | PR 1.39 (1.06–1.83) |
| De Socio et al., 2020 | BMI > 25 (vs <25) | OR 0.69 (0.50–0.96) |
| Elf et al., 2018 (men) | BMI (continuous) | OR 0.90 (0.80–0.90) |
|  | **Health conditions** |  |
| De Socio et al., 2020 | ASCVD (by one point) | OR 1.13 (1.08–1.17) |
| De Socio et al., 2020 | Cough (COPD symptoms) (vs No) | OR 25.93 (10.32–65.19) |
| De Socio et al., 2020 | Dyslipidaemia (vs No) | OR 1.44 (1.04–2.01) |
| De Socio et al., 2020 | Dyspnoea (COPD symptoms) (vs No) | OR 2.77 (1.40–5.47) |
| De Socio et al., 2020 | Hepatitis C virus positive (vs No) | OR 2.79 (1.68–4.63) |
| De Socio et al., 2020 | Sputum production (COPD symptoms) (vs No) | OR 31.11 (9.68–100.10) |
| Miles et al., 2019 (women) | Asthma (vs No) | OR 1.98 (1.05–3.71) |
| Regan et al., 2016 | CVD risk (vs No) | OR 0.76 (0.69–0.83) |
| Torres et al., 2014 | Cancer | OR 0.46 (0.25–0.83) |
|  | **HIV-related factors** |  |
| Akhtar-Khaleel et al., 2016a | Detectable viral load (vs No) | PR 1.04 (1.02–1.06) |
| Amiya et al., 2011 | Bothersome HIV symptoms | OR 2.06 (1.04–4.10) |
| Bhata et al., 2018 | Sexual contact last 3 months (vs No) | OR 3.14 (1.02–9.74) |
| Bhata et al., 2018 | HIV infection pathway of sex workers (vs Others) | OR 15.2 (4.35–53.8) |
| Regan et al., 2016 | HIV RNA >400 copies/ml (vs ≤400) | OR 1.56 (1.25–1.99) |
| Torres et al., 2014 | HIV infection pathway of IDU (vs Heterosexuals) | OR 2.89 (1.24–6.67) |
| Torres et al., 2014 | HIV infection pathway of MSM (vs Heterosexuals) | OR 2.36 (1.87–2.98) |
| Torres et al., 2014 | HIV infection pathway of other than MSM & IDU (vs Heterosexuals) | OR 1.37 (1.07–1.75) |
| Ompad et al., 2014 | Gastrointestinal opportunity infection (vs No) | OR 2.65 (1.07–6.60) |
| Ompad et al., 2014 | Detectable viral load (switched direction of Undetectable viral load) (vs No) | OR 3.13 (1.23–7.69) |
|  | **Psychosocial factors** |  |
| De Socio et al., 2020 | Psychiatric comorbidity (vs No) | OR 2.89 (1.55–5.39) |
| Gamarel et al., 2020 | Future orientation score | OR 0.84 (0.45–0.97) |
| Gamarel et al., 2020 | Internalised HIV Stigma score | OR 1.33 (1.03–1.72) |
| Mdege et al., 2021 | Mean EQ5D-3L Score (HRQoL) | OR 5.25 (1.18–23.35) |
| Mdege et al., 2021 | Perceived stress score | OR 2.23 (1.50–3.34) |
| Mdodo et al., 2015 | Major depression (vs No) | PD 11.20 (5.20–17.20) |
| Regan et al., 2016 | Mood disorders (vs No) | OR 1.93 (1.64–2.31) |
| Reisen et al., 2011 | Attitudes concerning adverse effects (vs No) | OR 0.43 (0.26–0.74) |
| Stewart et al., 2011 | Depression disorder (vs No) | OR 1.88 (1.12–3.16) |
| Teixeira et al., 2020 | Anxiety or depression (vs No) | OR 2.15 (1.26–3.68) |
| Teixeira et al., 2020 | Religious belief (yes) | OR 2.18 (1.06–4.50) |
| Zhang et al., 2018 | Enacted stigma score | OR 1.37 (1.05–1.78) |
|  | **Partner-related factors** |  |
| Bhata et al., 2018 | Having HIV+ spouse (vs No) | OR 0.10 (0.04–0.27) |
| Brath et al., 2016 | Partner smokes daily (vs Doesn't smoke) | OR 8.78 (4.49–17.17) |
| Mdege et al., 2021 | More than 2 smokers among 5 closest friends (vs No one) | OR 3.97 (2.08–7.59) |
|  | **Substance abuse** |  |
| Akhtar-Khaleel et al., 2016a | Marijuana use past 6 months | PR 1.09 (1.05–1.13) |
| Cropsey et al., 2016 | Prior substance use last 3 months (vs Never) | OR 6.62 (4.98–8.8) |
| de Dios et al., 2016 | Nicotine patch adherence (No) | OR 0.63 (0.47–0.83) |
| de Dios et al., 2016 | Nicotine dependence (No) | OR 1.28 (1.04–1.58) |
| De Socio et al., 2020 | Prior alcohol use (vs No) | OR 6.01 (1.31–27.55) |
| Edwards et al., 2020 | Cannabis occasional use (vs No) | OR 2.41 (1.71–3.67) |
| Edwards et al., 2020 | Cannabis regular use (vs No) | OR 6.24 (3.60–10.82) |
| Lam et al., 2020 | Substance use disorder | PR 1.67 (1.42–1.95) |
| Lam et al., 2020 | Tobacco use disorder | PR 12.4 (10.8–14.2) |
| Mdege et al., 2021 | Problem alcohol use | OR 3.96 (2.34–6.71) |
| Mdodo et al., 2015 | Binge drinking (vs None) | PD 13.50 (8.30–18.70) |
| Mdodo et al., 2015 | Drug use (vs No) | PD 20.50 (15.20–25.80) |
| Miles et al., 2019 (men) | Prior drug use (vs Never) | OR 1.45 (1.26–1.68) |
| Miles et al., 2019 (men) | Prior marijuana use (vs Never) | OR 1.17 (1.04–1.31) |
| Pacek et al., 2014a | Illicit drug use (vs No) | OR 2.90 (1.58–5.30) |
| Pacek et al., 2014b | Substance abuse treatment (No) | OR 26.21 (4.37–157.50) |
| Reisen et al., 2011 | Alcohol & drug use | OR 2.24 (1.08–4.65) |
| Shirley et al., 2013 | Past inhalant drug use | OR 3.10 (1.40–7.00) |
|  |  |  |
| *ASCVD: Atherosclerotic Cardiovascular Disease, BMI: Body mass index, CVD: Cardiovascular disease, HIV: Human immunodeficiency virus, HRQoL: Health-related quality of life, IDU: Illicit drug use, MSM: Men who have sex with men, OR: Odds ratio, PD: Prevalence difference, PR: Prevalence ratio, RNA: Ribonucleic acid, SES: Socioeconomic status* | | |

| Study Reference | Type of outcomes | Associated factor of smoking abstinence | OR (95% CI) |
| --- | --- | --- | --- |
|  |  | **Sociodemographic factors** |  |
| Aigner et al., 2017 | CO7DPPA-e12M | Age | 0.97 (0.89–1.06) |
| Humfleet et al., 2013 | CO7DPPA-e13M | Employed (vs Unemployed) | 0.68 (0.49–0.93) |
| Moadel et al., 2012 | CO7DPPA-e3M | Latino ethnicity | 4.11 (1.06–16.00) |
| Shutter et al., 2020 | CO7PPA-e13M | Latino ethnicity | 0.31 (0.09–1.08) |
| Shutter et al., 2020 | CO7PPA | Latino ethnicity | 0.45 (0.22–0.95) |
| Stanton et at., 2020 | CO7DPPA-3M | Transitional housing or homelessness (vs No) | 0.10 (0.01–0.50) |
| Stanton et at., 2020 | CO7DPPA-3M | Other ethnicity than White (vs Black) | 0.32 (0.10–0.85) |
| Stanton et at., 2020 | CO7DPPA-6M | Education ≥high school graduation (vs No) | 2.63 (1.15–6.59) |
| Taniguchi et al., 2020 | CO2wPPA | Age | 1.04 (1.00–1.08) |
| Zyambo et al., 2019 | SRA | Age (10-year interval) | 0.06 (0.02–0.27) |
|  |  | **Health-related factors** |  |
| Aigner et al., 2017 | CO7DPPA-e12M | Pain | 1.01 (1.00–1.03) |
| Buchberg et al., 2016 | CO7DPPA-3M | BMI decrease 7-day (<−0.41 units) | 4.22 (1.65–10.82) |
| Buchberg et al., 2016 | CO7DPPA-3M | BMI increase 7-day (>0.81 units) | 4.22 (1.62–11.01) |
| Huber et al., 2012 | 12MSRA | Cardiovascular event during the last 2 years (vs No) | 2.29 (1.67–3.14) |
| Miles et al., 2019 (women) | SRA | Asthma | 3.92 (1.10–13.76) |
| Stanton et at., 2020 | CO7DPPA-6M | Distress tolerance score | 0.46 (0.27–0.73) |
| Zyambo et al., 2019 | SRA | Duration of care (year) | 0.96 (0.92–0.99) |
| Zyambo et al., 2019 | SRA | Public insurance (vs Private) | 0.66 (0.44–0.96) |
| Zyambo et al., 2019 | SRA | Uninsured (vs Private) | 0.70 (0.50–0.99) |
|  |  | **Risky behavioural factors** |  |
| Huber et al., 2012 | SRA-6M | Moderate alcohol use (vs None) | 0.77 (0.63–0.95) |
| Shutter et al., 2020 | CO7DPPA-e13M | Current marijuana use (vs No) | 2.18 (1.03–4.60) |
| Shutter et al., 2020 | CO7DPPA-e13M | Cocaine use (vs No) | 0.22 (0.05–0.94) |
| Zyambo et al., 2019 | SRA | Current substance use (vs Never) | 0.56 (0.31–0.96) |
|  |  | **HIV-related factors** |  |
| Ashare et al., 2019 | CO7DPPA-3M | ART adherence (vs No) | 1.01 (1.00–1.02) |
| Huber et al., 2012 | SRA-6M | HIV infection pathway of former IDU (vs Heterosexuals) | 0.55 (0.46–0.65) |
| Huber et al., 2012 | SRA-6M | HIV infection pathway of current IDU (vs Heterosexuals) | 0.25 (0.17–0.37) |
| Shutter et al., 2020 | CO7DPPA-e13M | HIV viral load ≥ 40 copies/mL (vs No) | 0.32 (0.11–0.93) |
| Stanton et at., 2020 | CO7DPPA-3M | HIV viral load ≥40 copies/m | 0.37 (0.13–0.91) |
| Zyambo et al., 2019 | SRA | HIV RNA < 200 copies/ml | 1.39 (1.04–1.88) |
|  |  | **Smoking-related factors** |  |
| Aigner et al., 2017 | CO7DPPA-e12M | FTND scale | 0.59 (0.45–0.76) |
| Ashare et al., 2019 | CO7DPPA-3M | Nicotine metabolism (vs No) | 3.08 (1.01–9.37) |
| Browning et al., 2016 | CO7DPPA-3M | High participant responsiveness (vs Low) | 8.16 (3.36–19.82) |
| Browning et al., 2016 | CO7DPPA-3M | Counselling adherence (vs No) | 7.58 (2.78–20.66) |
| Browning et al., 2016 | CO7DPPA-12M | High participant responsiveness (vs Low) | 4.33 (1.51–12.44) |
| Browning et al., 2016 | CO7DPPA-12M | Counselling adherence (vs No) | 5.91 (1.66–21.02) |
| De Socio et al., 2020 | 1MSRA | Use of drugs/e-cigarettes for smoking cessation (vs No) | 7.59 (3.29–19.94) |
| Huber et al., 2012 | 12MSRA | Duration of intervention (year) | 1.24 (1.07–1.45) |
| Humfleet et al., 2013 | CO7DPPA-e13M | Completers (vs non-Completers) | 13.19 (1.28–136.20) |
| Shutter et al., 2020 | CO7PPA | Non-daily smokers | 3.30 (1.73–6.30) |
| Shutter et al., 2020 | CO7PPA-e13M | Non-daily smokers | 2.85 (1.25–6.51) |
| Stanton et at., 2015 | CO7DPPA-6M | Square root (NRT dose) (in multiples of 7 mg × 7 days) | 1.45 (1.08–1.96) |
| Stanton et at., 2015 | CO7DPPA-6M | Daily smokers | 0.23 (0.08–0.66) |
| Stanton et at., 2020 | CO7DPPA-3M | Nicotine dependence (vs No) | 0.81 (0.69–0.95) |
| Stanton et at., 2020 | CO7DPPA-6M | Any use of nicotine replacement therapy before enrolment (vs No) | 0.42 (0.20–0.88) |
|  |  | **Smoking cessation-related behavioural factors** |  |
| Chew et al., 2014 | CO7DPPA-6M | Preparation stage of change (vs Pre-contemplation and contemplation) | 8.26 (1.02–6.67) |
| De Socio et al., 2020 | 6MSRA | Preparation/action stage of change (vs Pre-contemplation) | 4.29 (1.44–12.06) |
| De Socio et al., 2020 | 1MSRA | Contemplation stage of change (vs Pre-contemplation) | 2.55 (1.03–6.13) |
| De Socio et al., 2020 | 1MSRA | Preparation/action stage of change (vs Pre-contemplation) | 4.57 (2.08–9.65) |
| Humfleet et al., 2013 | CO7DPPA-e13M | Desire to quit (vs No) | 1.72 (1.11–2.68) |
| Kim et al., 2020 | CO7DPPA-3M | Craving for cigarettes | 0.62 (0.49–0.78) |
| Stanton et at., 2020 | CO7DPPA-3M | Motivation to quit (vs No) | 1.43 (1.07–1.96) |
| Taniguchi et al., 2020 | CO2WPPA | Self-efficacy ≥80% (<80%) | 9.99 (1.86–53.73) |
| Triant et al., 2020 | 30DSRA-6M | Confidence in quitting | 1.95 (1.37–2.78) |
| *ART: Anti-retroviral therapy, BMI: Body mass index, CO2wPPA: Carbon monoxide-verified 2-week point prevalence of abstinence, CO7DPPA-3/6/12M: Carbon monoxide-verified 7-day point prevalence of abstinence at 3/6/12 months, CO7DPPA-e3/12/13M: Carbon monoxide-verified 7-day point prevalence of abstinence at 3/12/13 months after end of treatment, SRA-6M: Self-reported abstinence at 6 months, 1M/6M/12MSRA: 1/6/12-month self-reported abstinence, 30DSRA-6M: 30-day self-reported abstinence at 6 months, FTND: Fagerström Test for Nicotine Dependence, HIV: Human immunodeficiency virus, IDU: Illicit drug use, NRT: Nicotine replacement therapy, OR: Odds ratio, RNA: Ribonucleic acid* | | | |

| Study Reference | Type of outcomes | Associated factor of other smoking cessation outcomes | Measure Est. (95% CI) |
| --- | --- | --- | --- |
| Sociodemographic factors | | | |
| Amiya et al., 2011 | Readiness to quit | Formal education (vs No) | OR 0.12 (0.02–0.71) |
| Browning et al., 2016 | Adherence to pharmacotherapy | Age | OR 1.06 (1.02–1.09) |
| Browning et al., 2016 | Adherence to pharmacotherapy | Other ethnicity (vs White) | OR 0.48 (0.28–0.83) |
| Browning et al., 2016 | Adherence to pharmacotherapy | More than high school educational level (vs less than high school) | OR 2.25 (1.07–4.70) |
| Browning et al., 2016 | Adherence to tobacco dependence treatment calls | Age | OR 1.05 (1.02–1.08) |
| Browning et al., 2016 | Adherence to tobacco dependence treatment calls | Other ethnicity (vs White) | OR 0.51 (0.30–0.84) |
| Browning et al., 2016 | Adherence to tobacco dependence treatment calls | More than high school educational level (vs less than high school) | OR 2.25 (1.20–5.31) |
| Cioe et al., 2017 | Intention to quit (future) | MSM of colour (vs No) | OR 11.63 (1.28–105.54) |
| Cioe et al., 2017 | Intention to quit (Immediate) | MSM of colour (vs No) | OR 11.63 (1.28–105.54) |
| Lam et al., 2020 | Use of cessation treatment in the last 2 years | Hispanic ethnicity (vs non-Hispanic White) | PR 0.57 (0.36–0.90) |
| McQueen et al., 2014 | Prior use of smoking cessation aids | Previously married (vs Never married) | OR 0.33 (0.11–0.98) |
| McQueen et al., 2014 | Prior use of smoking cessation aids | Just enough income (vs Not) | OR 2.79 (1.34–5.84) |
| Nguyen et al., 2015 | Readiness to quit | Moderate income (vs Lowest) | OR 2.20 (1.33–3.62) |
| Nguyen et al., 2015 | Readiness to quit | Highest income (vs Lowest) | OR 1.84 (1.09–3.10) |
| Pacek et al., 2014c | Lifetime use of NRT/meds | Age 54-65 (vs 28-44) | OR 3.38 (1.57–7.26) |
| Pacek et al., 2014c | Lifetime use of NRT/meds | Age 45-49 (vs 28-44) | OR 2.70 (1.19–6.11) |
| Pacek et al., 2014c | Lifetime use of NRT/meds | White (vs Black) | OR 3.56 (1.2–10.62) |
| Pacek et al., 2014c | Interest in quitting | Age 54-65 (vs 28-44) | OR 4.64 (1.59–13.47) |
| Shahrir et al., 2020 | Quitting attempt | Other ethnicity than Hispanic & Black (vs White) | OR 2.62 (1.04–6.56) |
| Shutter et al., 2014 | More interactive clicks | High school graduation (vs No) | OR 4.08 (1.02–16.4) |
| Shutter et al., 2014 | More time spent logged into site | Anxiety score | OR 1.17 (1.04–1.31) |
| Tseng et al., 2017 | Adherence to Varenicline | Baseline adherence self-efficacy | OR 2.20 (1.43–3.39) |
| Vijayaraghavan et al., 2014 | Quitting attempt | Age | OR 1.10 (1.00–1.20) |
| Health-related factors | | | |
| Barré et al., 2021 | Quitting attempt | Body mass index | OR 1.08 (1.00–1.19) |
| Barré et al., 2021 | Quitting attempt | HCV cure | OR 1.18 (1.08–4.35) |
| McQueen et al., 2014 | Prior use of smoking cessation aids | Self-rated health status (1=excellent to 5=poor) | OR 1.49 (1.06–2.08) |
| McQueen et al., 2014 | Prior use of smoking cessation aids | Medicaid insurance (vs Others) | OR 2.34 (1.06–5.16) |
| Pacek et al., 2018 | Risk perception | On HIV medications (vs No) | Coeff 0.65 (0.17–1.12) |
| Shahrir et al., 2016 | Receipt of Any Smoking Cessation Medication | Psychiatric disorders (vs No) | OR 1.80 (1.06–2.99) |
| Shahrir et al., 2016 | Receipt of Any Smoking Cessation Medication | Pulmonary disease (vs No) | OR 1.96 (1.06–3.61) |
| Shahrir et al., 2020 | Quitting attempt | Pulmonary disease | OR 2.77 (1.11–6.93) |
| Quinn et al., 2020 | Adherence to Varenicline | Baseline creatinine | OR 0.97 (0.96–0.99) |
| Risky behavioural factors | | | |
| Barré et al., 2021 | Quitting attempt | Cannabis use | OR 0.53 (0.28–1.00) |
| Barré et al., 2021 | Quitting attempt | Psychoactive substance use | OR 0.24 (0.07–0.99) |
| Browning et al., 2016 | Adherence to pharmacotherapy | Binge drinking at least monthly (vs No) | OR 0.54 (0.30–0.98) |
| Browning et al., 2016 | Adherence to tobacco dependence treatment calls | Binge drinking at least monthly (vs No) | OR 0.45 (0.24–0.82) |
| De Socio et al., 2020 | Quitting attempt | Use of drugs/e-cigarettes for smoking cessation (vs No) | OR 4.37 (1.72–10.65) |
| Nguyen et al., 2015 | Readiness to quit | Binge drinking (vs No) | OR 0.47 (0.25–0.89) |
| Nguyen et al., 2015 | Readiness to quit | Lifetime drug use (vs No) | OR 0.31 (0.14–0.67) |
| Nguyen et al., 2015 | Readiness to quit | Lifetime drug use & Binge drinking (vs No) | OR 0.40 (0.20–0.77) |
| Nguyen et al., 2015 | Readiness to quit | Current Methadone use (vs No) | OR 0.40 (0.17–0.94) |
| Shahrir et al., 2020 | Quitting attempt | Unhealthy alcohol use (vs No) | OR 0.70 (0.53–0.92) |
| Shutter et al., 2014 | More interactive clicks | Homosexual contact (vs No) | OR 0.26 (0.08–0.83) |
| Vijayaraghavan et al., 2014 | Quitting attempt | Illicit drug use in the past 90 days (vs No) | OR 0.20 (0.10–0.60) |
| Pain-related factors | | | |
| LaRowe et al., 2020 | Intention to quit | Pain intensity score | OR 1.28 (1.06–1.54) |
| LaRowe et al., 2020 | Intention to quit | Pain self-efficacy score | OR 1.05 (1.00–1.11) |
| Nguyen et al., 2015 | Readiness to quit | Current pain (vs No) | OR 1.60 (1.02–2.52) |
| Tobacco dependence | | | |
| Browning et al., 2016 | Adherence to pharmacotherapy | 11-20 cigarettes per day (vs <10) | OR 2.12 (1.12–4.00) |
| Cioe et al., 2017 | Intention to quit (future) | Intermittent smokers (vs Daily smokers) | OR 5.15 (1.67–15.94) |
| De Socio et al., 2020 | Quitting attempt | FTND score (by -1 point) | OR 1.26 (1.11–1.39) |
| McQueen et al., 2014 | Prior use of smoking cessation aids | FTND score | OR 1.21 (1.03–1.43) |
| Pacek et al., 2014c | Interest in quitting | Lifetime NRT/med use (vs No) | OR 2.02 (1.08–3.80) |
| Pacek et al., 2017 | Interest in quitting | Daily smoking (vs No) | OR 0.49 (0.30–0.80) |
| Shahrir et al., 2020 | Quitting attempt | 10-20 cigarettes per day (vs <10) | OR 0.74 (0.56–0.98) |
| Shapiro et al., 2011 | Intention to quit | Age at smoking initiation | OR 0.90 (0.82–0.98) |
| Shapiro et al., 2011 | Intention to quit | A recent quit attempt | OR 3.38 (1.64–6.96) |
| Vijayaraghavan et al., 2014 | Quitting attempt | Time to first cigarette in the morning <30 min (vs No) | OR 0.20 (0.10–0.70) |
| Smoking cessation-related psychosocial factors | | | |
| Amiya et al., 2011 | Readiness to quit | Presence of smoker among other household members (vs No) | OR 2.91 (1.16-7.29) |
| Browning et al., 2016 | Quitting attempt | Adherence to 80% or more of smoking cessation calls | OR 2.41 (1.01–5.77) |
| de Dios et al., 2016 | Adherence to nicotine patch | Social support frequency score | OR 1.14 (1.02–1.27) |
| McQueen et al., 2014 | Prior use of smoking cessation aids | Belief about harms of meds (Beliefs about Medicines Questionnaire) | OR 0.71 (0.51–0.98) |
| McQueen et al., 2014 | Prior use of smoking cessation aids | Social support for using meds (1 = not at all true to 5 = extremely true) | OR 1.31 (1.04–1.66) |
| Pacek et al., 2014c | Lifetime use of NRT/meds | Supporter lifetime for NRT/med use (vs No) | OR 2.13 (1.05–4.29) |
| Pacek et al., 2017 | Interest in quitting | Often/always have cessation discussions (vs Never/rarely) | OR 3.10 (1.64–5.86) |
| Shutter et al., 2014 | Visited all webpages | Anxiety score | OR 1.15 (1.01–1.31) |
| Shahrir et al., 2020 | Quitting attempt | Moderate to severe depression (PHQ-9) | OR 1.54 (1.15–2.07) |
| Quinn et al., 2020 | Adherence to Varenicline | Reduction in Anxiety symptoms (vs No) | OR 1.36 (1.09–1.69) |
| Quinn et al., 2020 | Adherence to Varenicline | Reduction in Insomnia (vs No) | OR 3.28 (1.04–10.33) |
| Smoking cessation-related behavioural factors | | | |
| Amiya et al., 2011 | Readiness to quit | Prior quit attempt(s) last 12 months (vs No) | OR 3.77 (1.48–9.58) |
| Donnelly et al., 2021 | Readiness to quit | Self-efficacy score | Coeff 0.51 (0.13–0.89) |
| De Socio et al., 2020 | Quitting attempt | Contemplation stage of change (vs Pre-contemplation) | OR 2.73 (1.32–5.49) |
| De Socio et al., 2020 | Quitting attempt | Preparation/action stage of change (vs Precontemplation) | OR 4.89 (2.65–8.8) |
| Shahrir et al., 2016 | Receipt of Any Smoking Cessation Medication | Contemplation to quit (vs No) | OR 3.42 (1.21–9.61) |
| Shapiro et al., 2011 | Intention to quit | A recent quit attempt (vs No) | OR 3.38 (1.64–6.96) |
| Pacek et al., 2017 | Interest in quitting | Lifetime quit attempt(s) (vs No) | OR 9.02 (4.80–17.00) |
| Vijayaraghavan et al., 2014 | Quitting attempt | Expect to quit in the next 1–6 months (vs No) | OR 4.30 (1.20–16.00) |
| Role of healthcare providers | | | |
| Amiya et al., 2011 | Readiness to quit | Smoking status assessment by a physician last 12 months (vs No) | OR 3.34 (1.05–10.61) |
| McQueen et al., 2014 | Prior use of smoking cessation aids | Physician prescription of meds (vs No) | OR 3.73 (1.70–8.16) |
| Pacek et al., 2017 | Interest in quitting | Provider encouragement to quit (vs No) | OR 3.37 (1.61–7.05) |
| Pacek et al., 2018 | Risk perceptions | Provider recommended quitting (vs No) | Coeff 1.04 (0.42–1.67) |
| *Coeff: Coefficient, FTND: Fagerström Test for Nicotine Dependence, MSM: Men who have sex with men, NRT: Nicotine replacement therapy, OR: Odds ratio, PR: Prevalence ratio,* | | | |


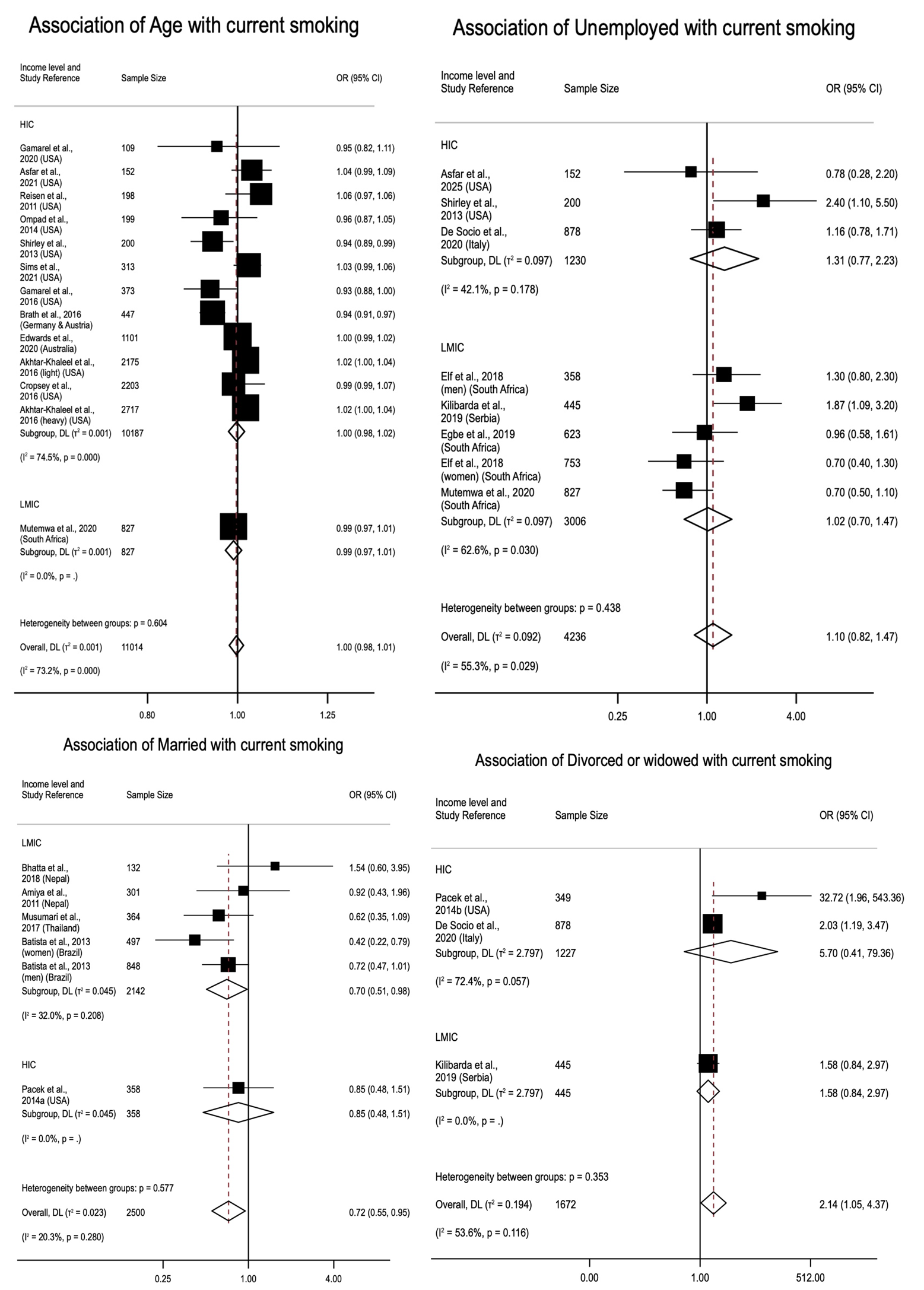
Figure S1 Forest plots of factors influencing current smoking by country income level (studies using Logistic regression)


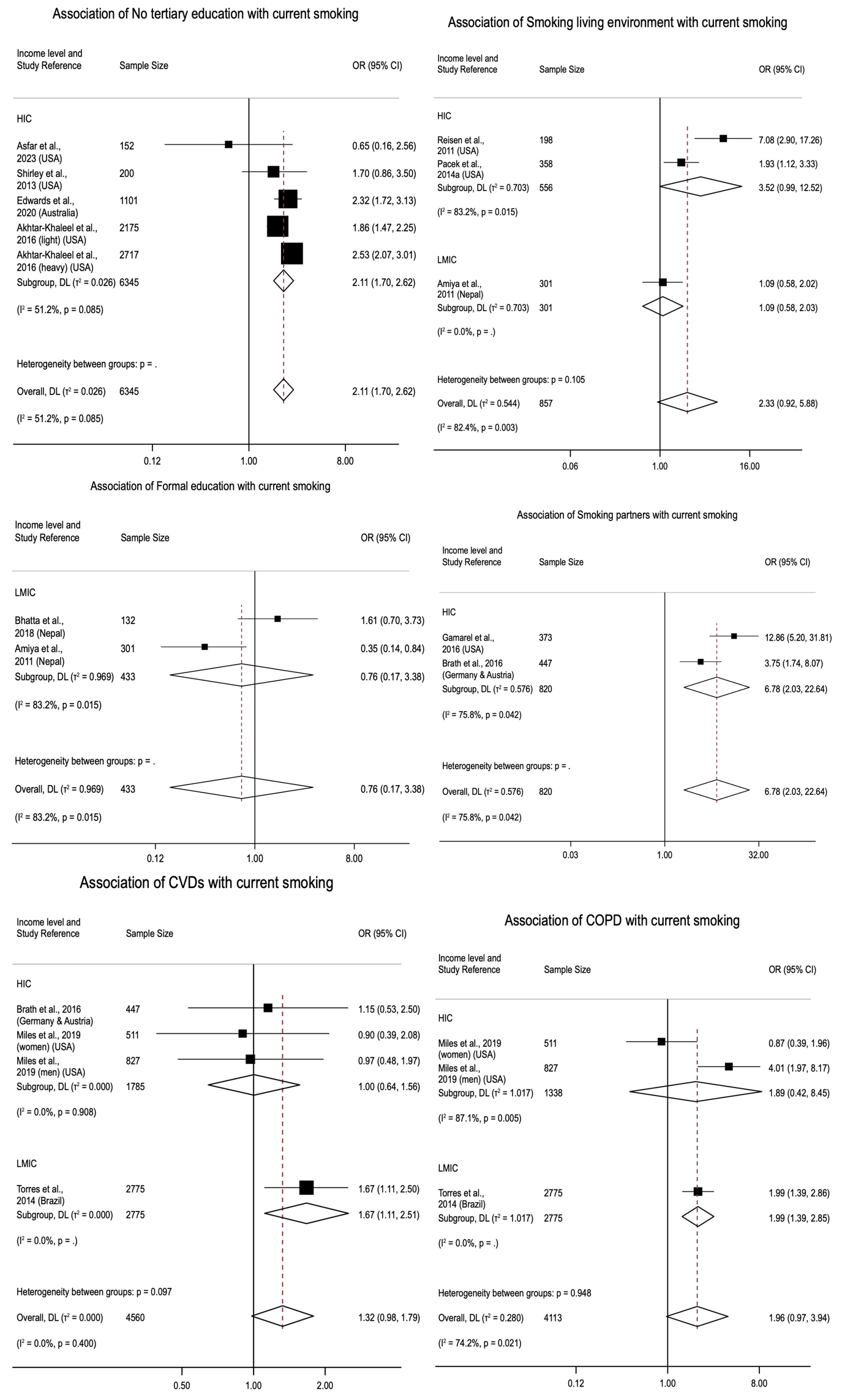

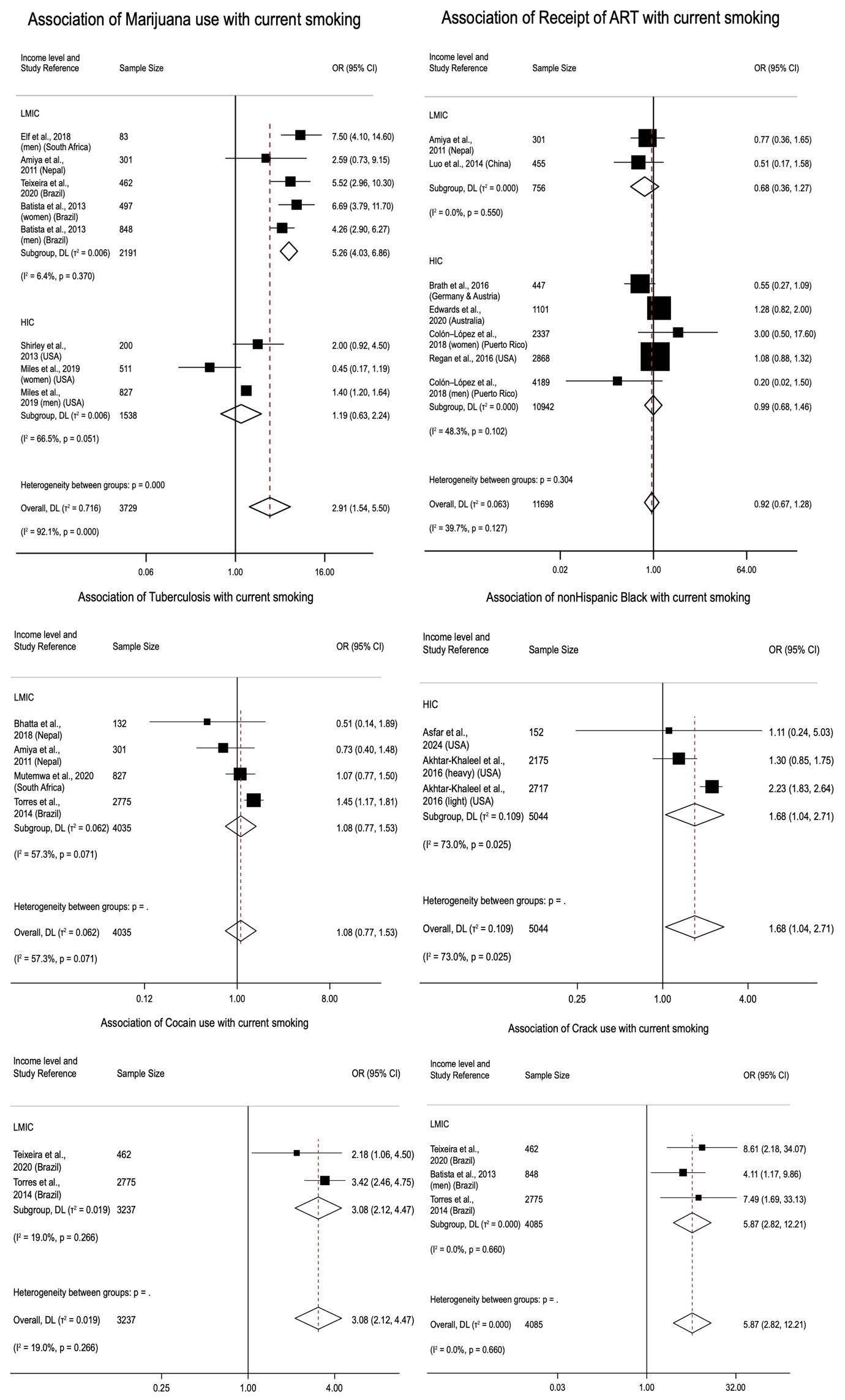

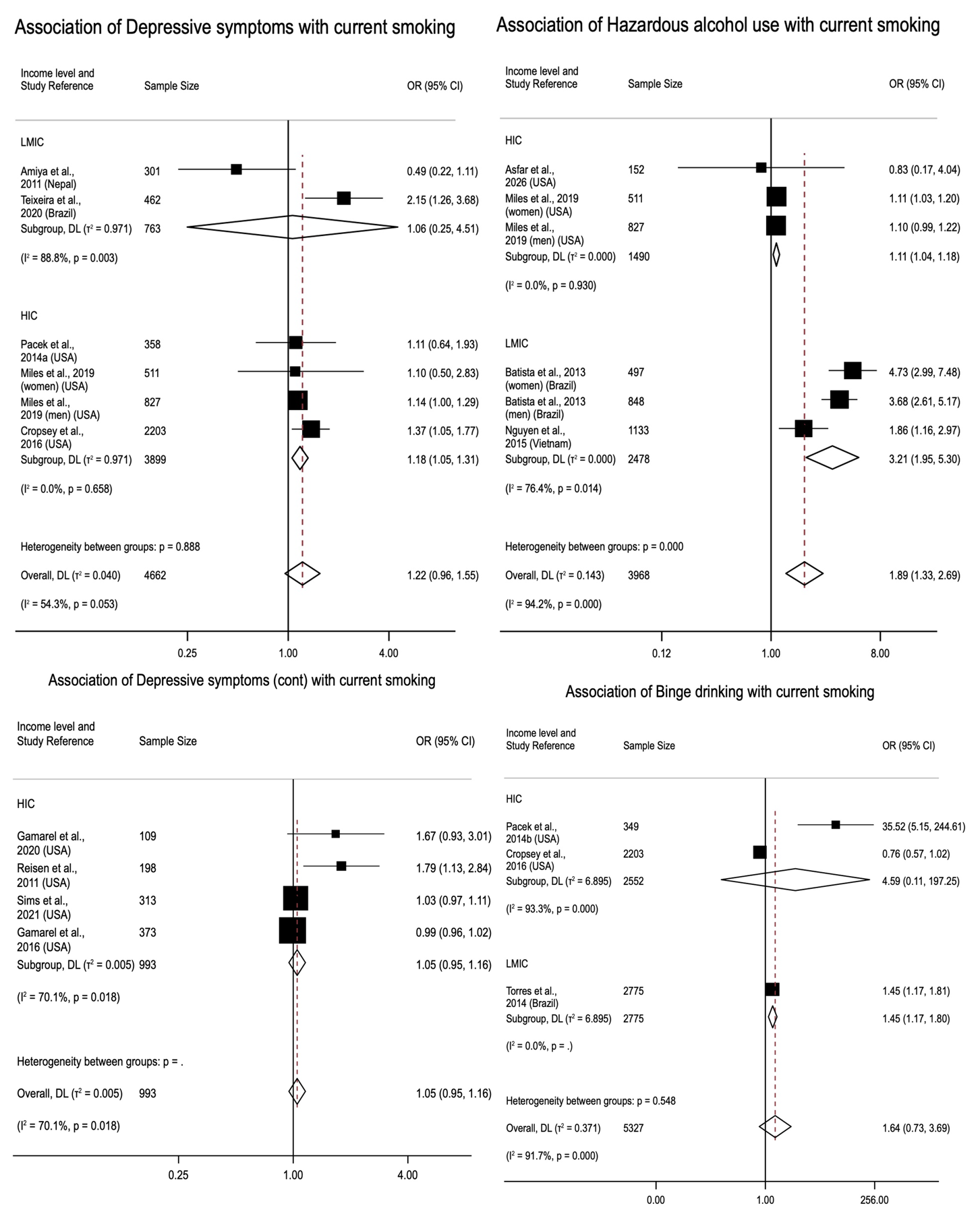


Figure S2 Forest plots of factors influencing current smoking (studies using Multilevel & Poisson regression)

**MULTILEVEL LOGISTIC REGRESSION**


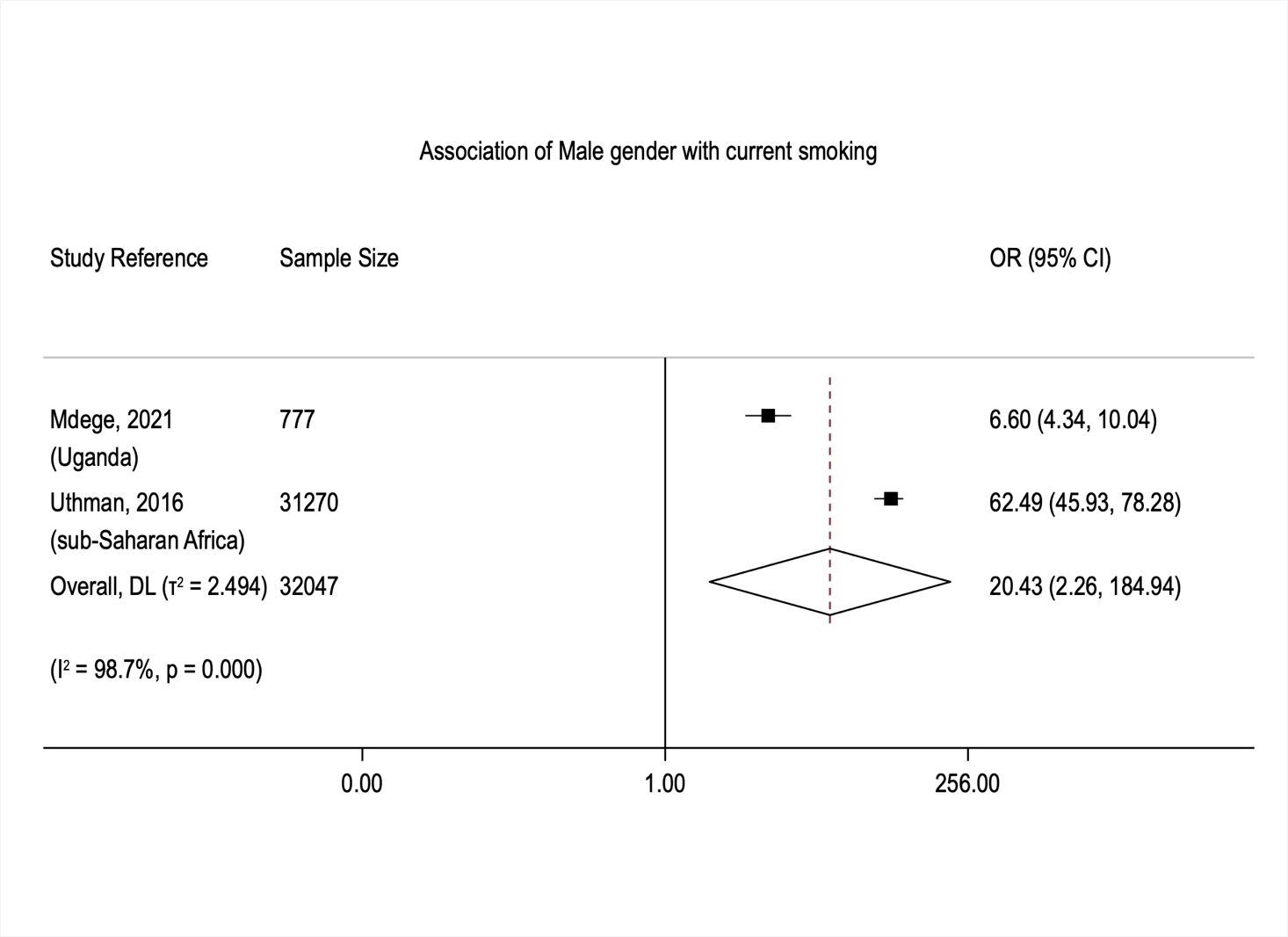


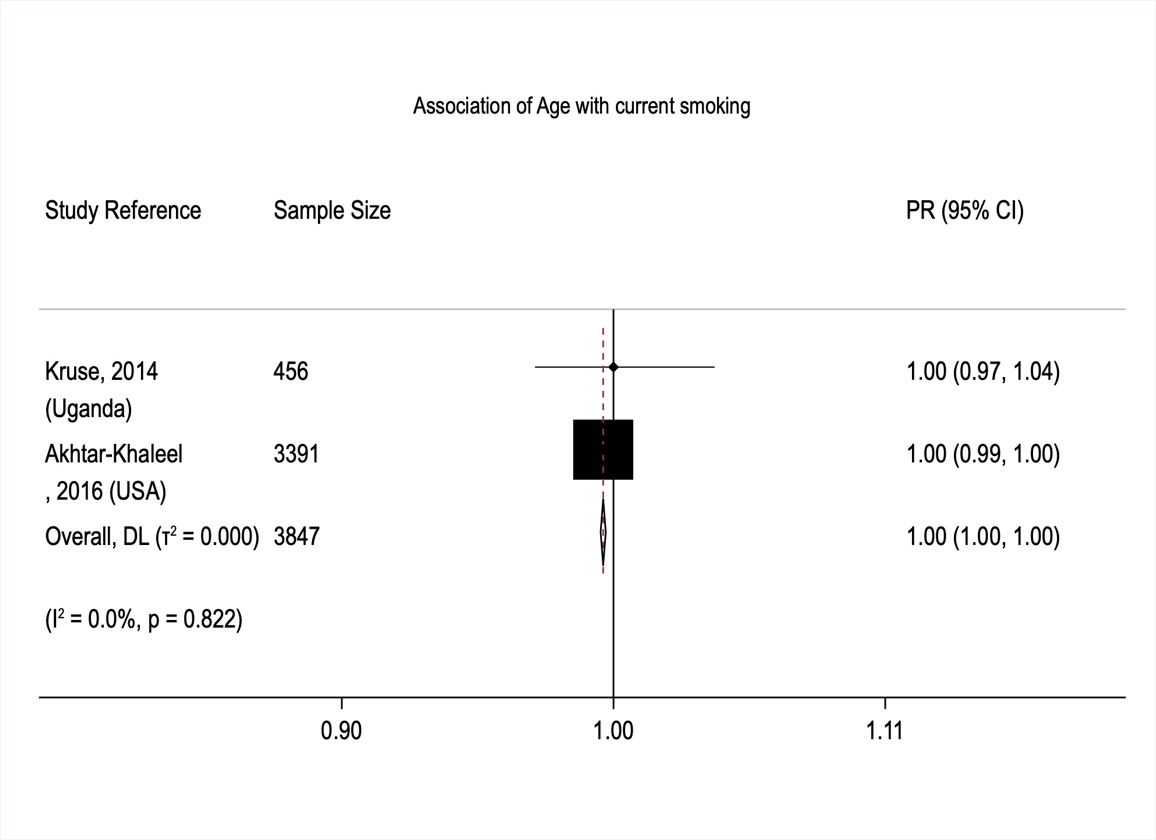
***POISSON REGRESSION***
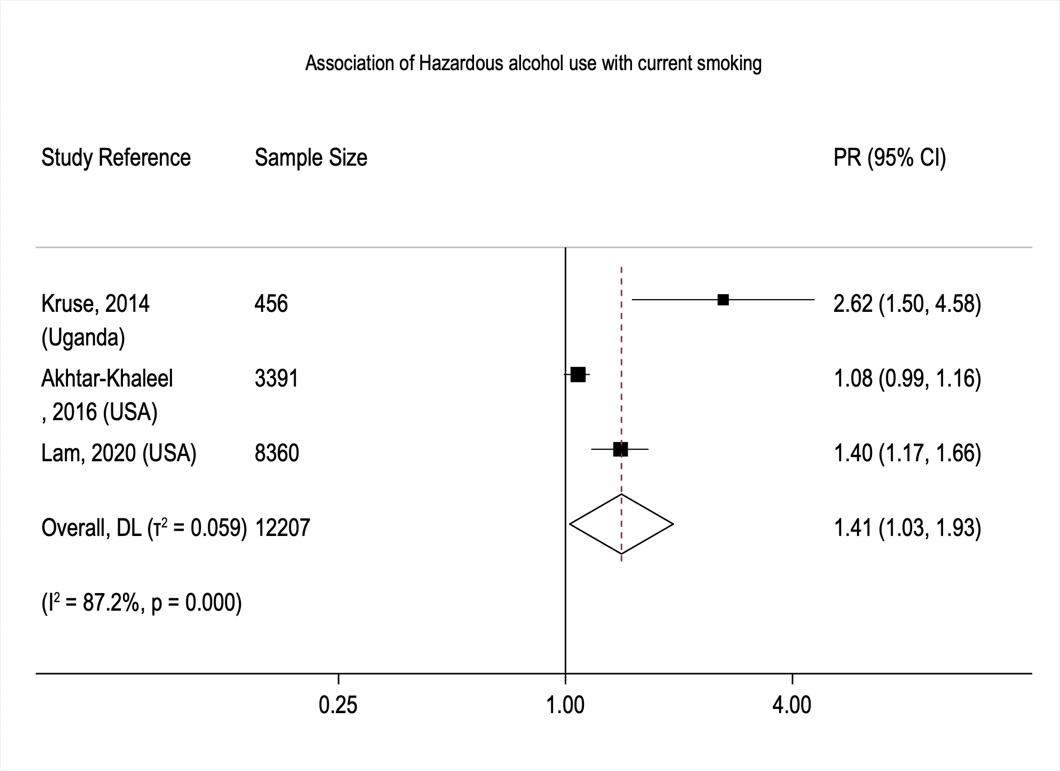

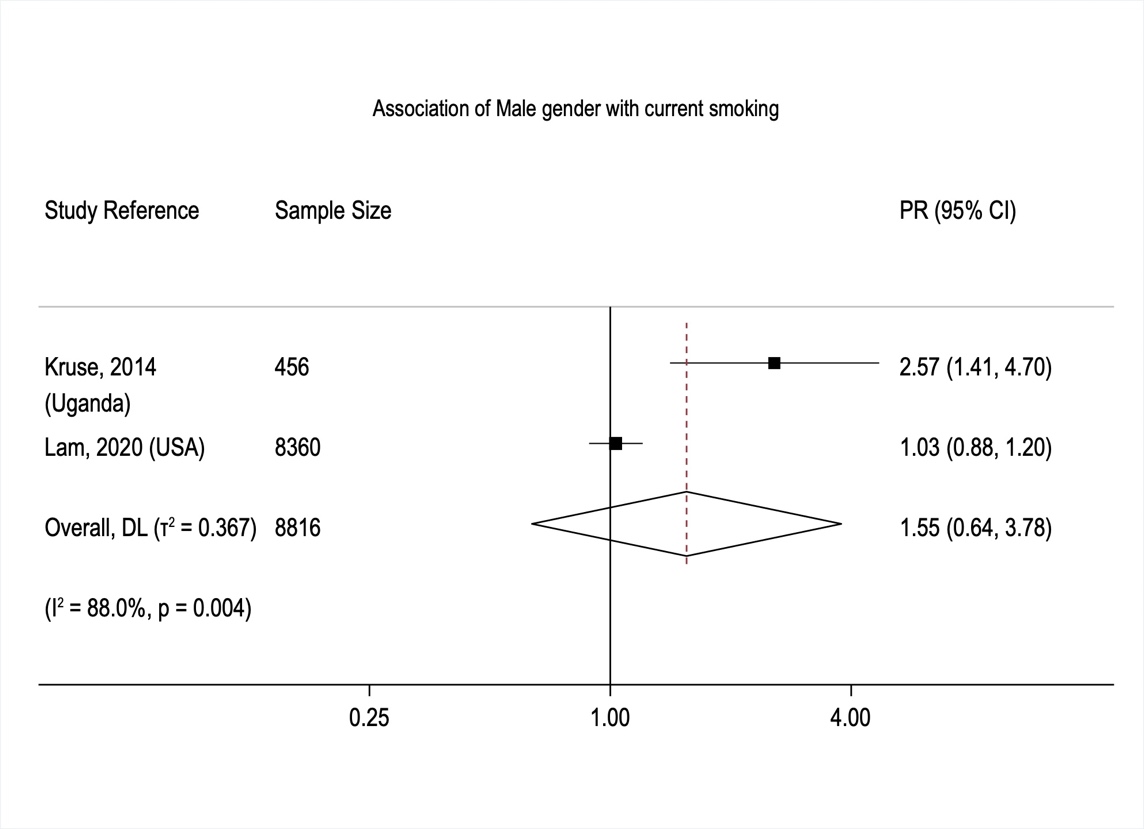

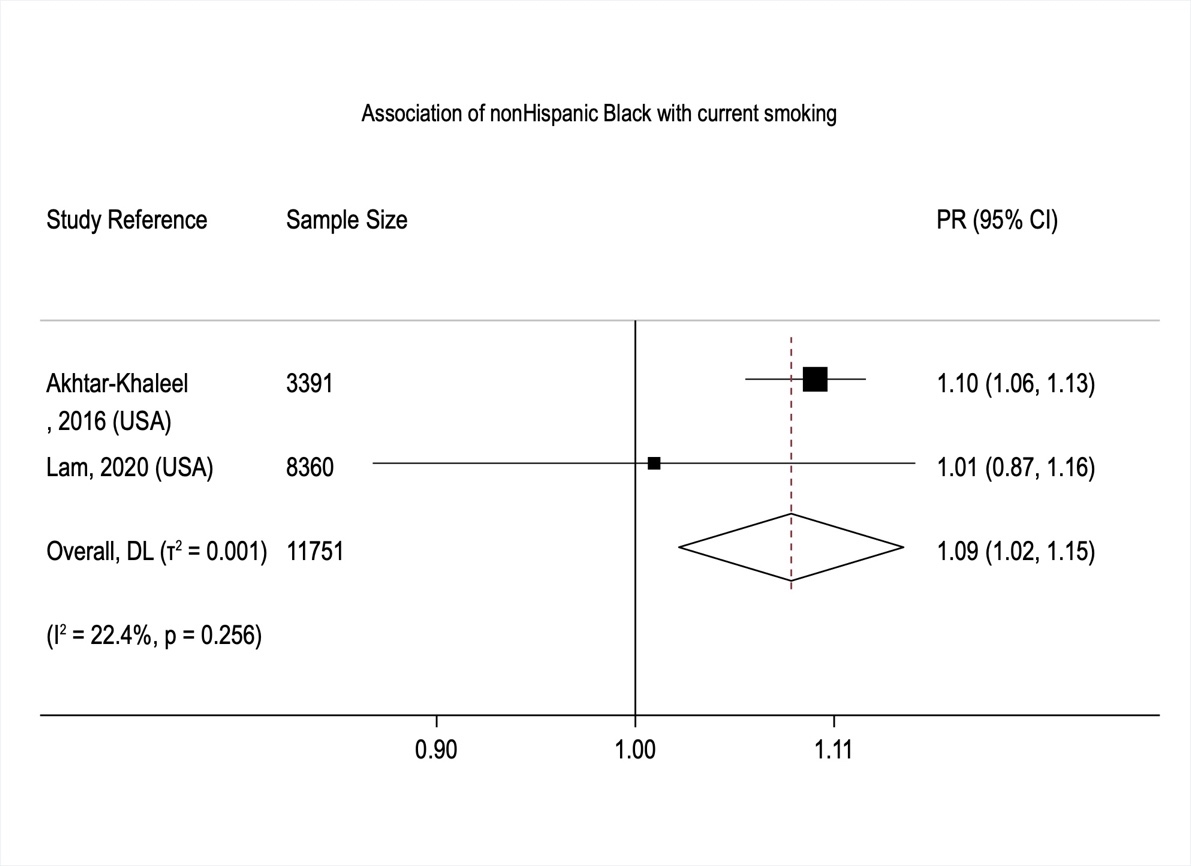


Figure S3 Forest plots of factors influencing smoking abstinence


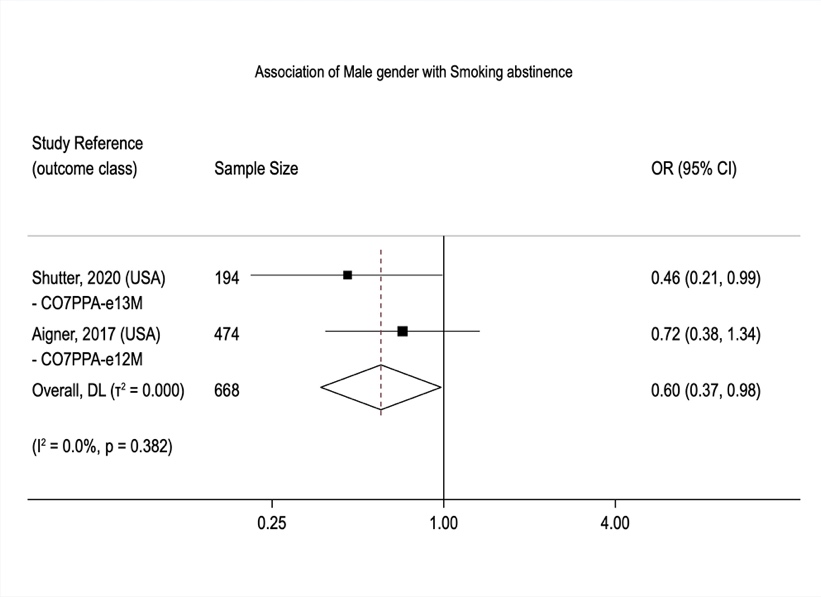

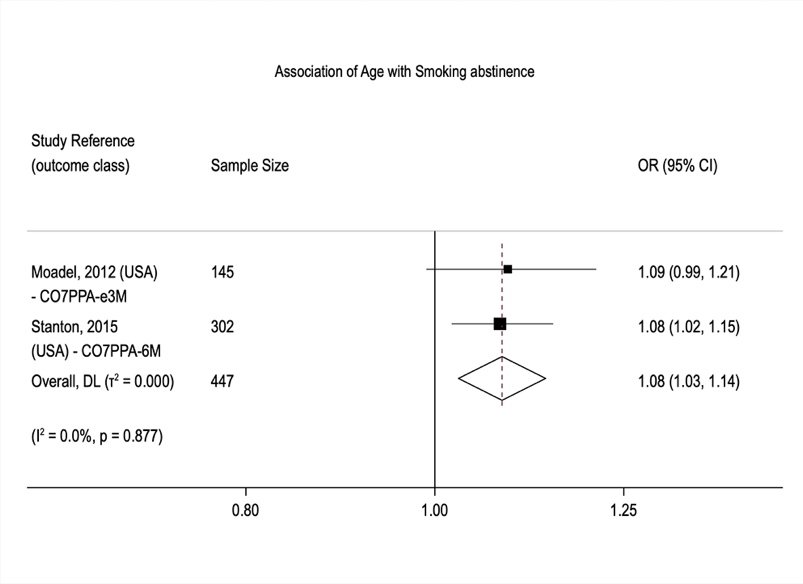

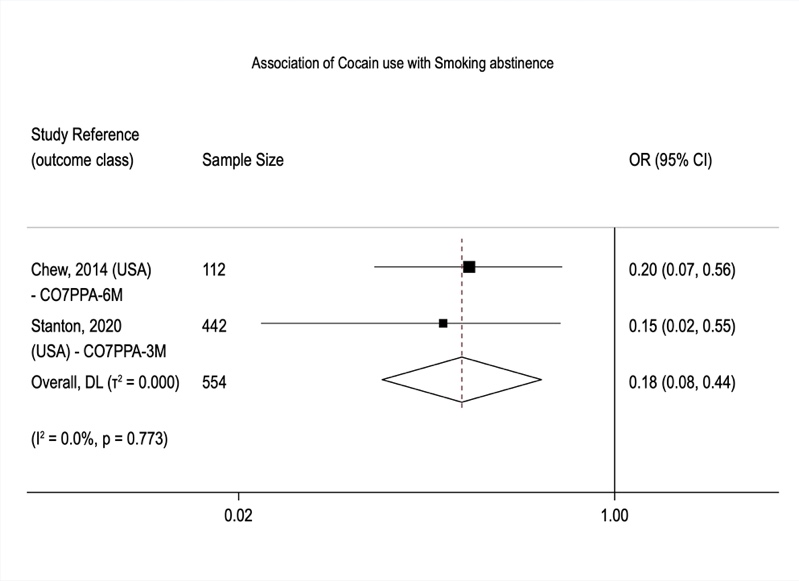

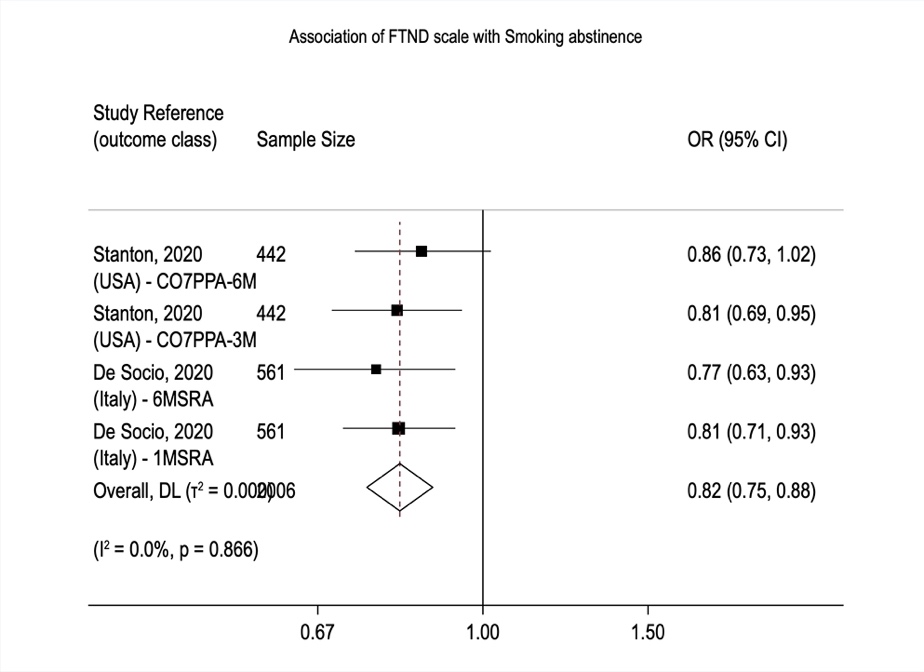

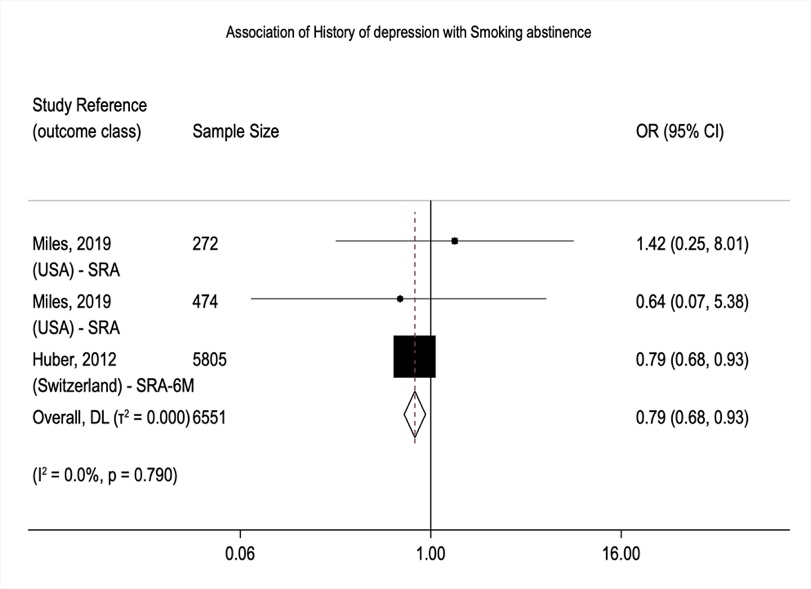

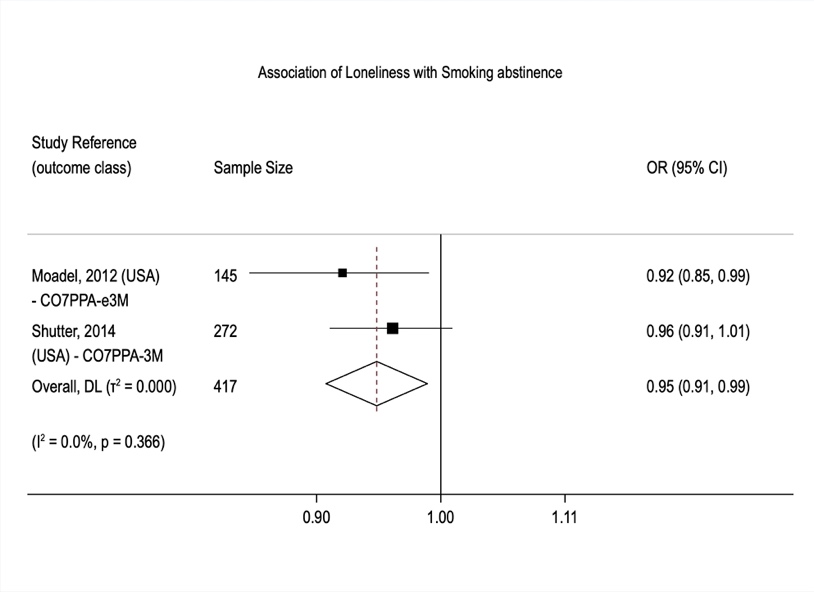

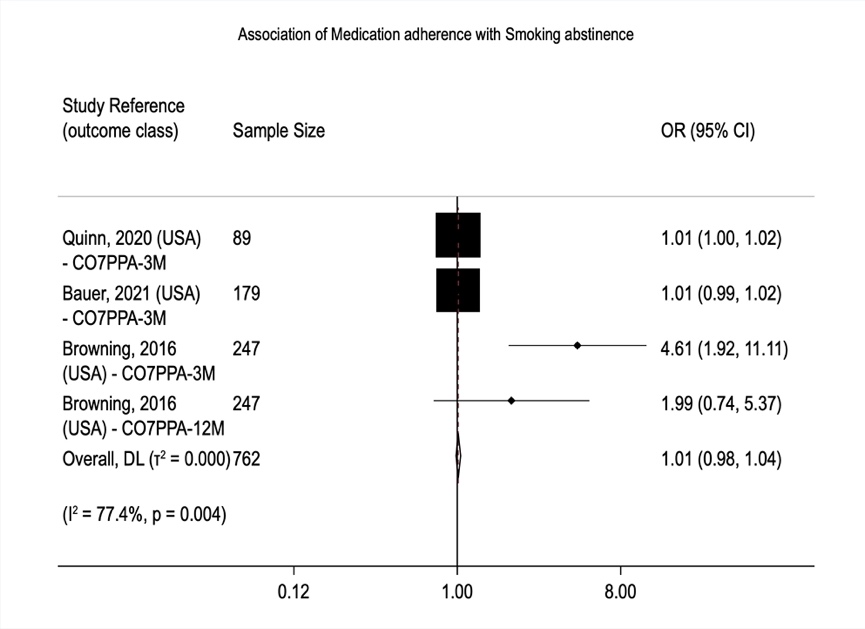

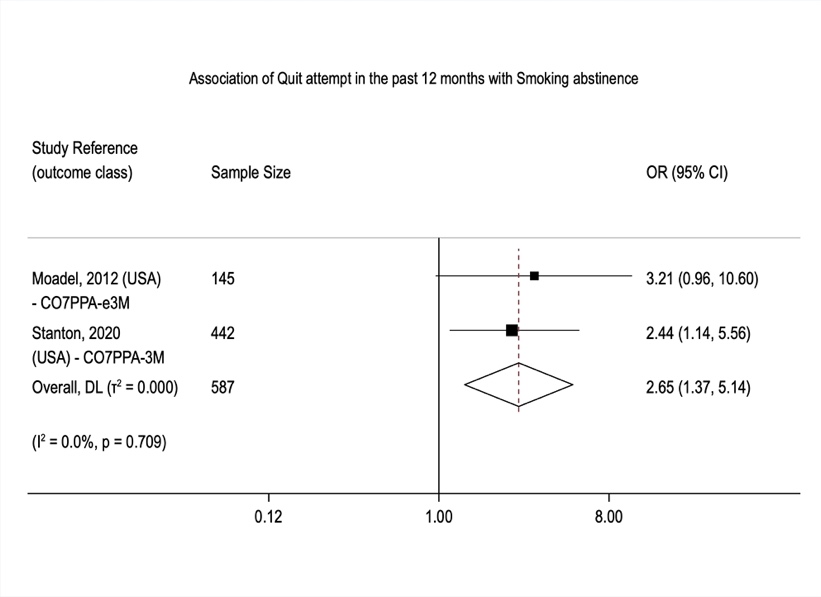

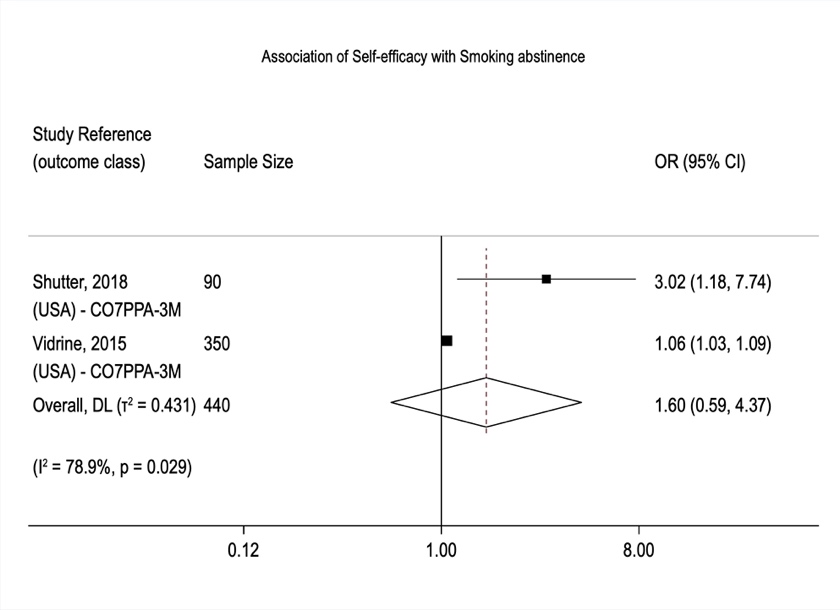

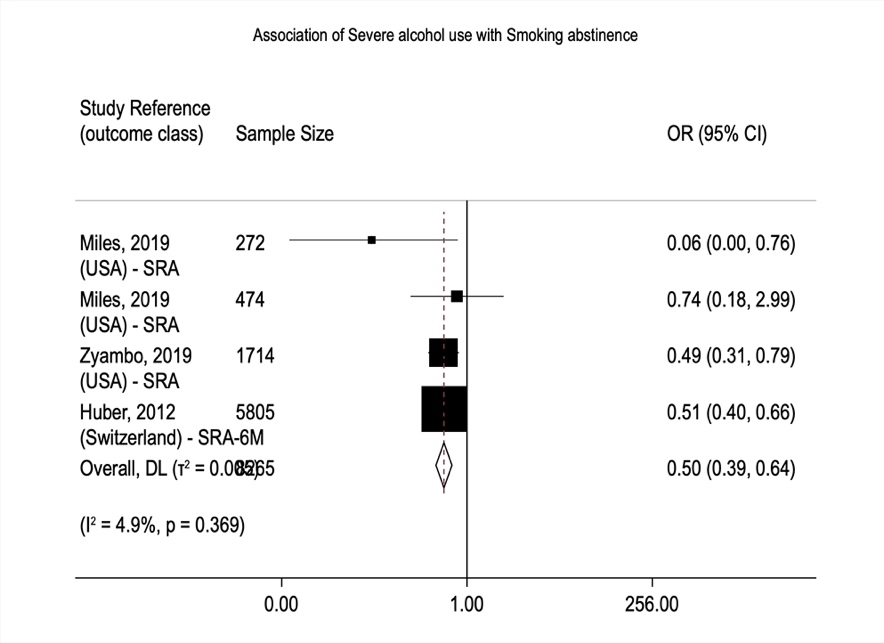

Supplement: Supplementary file 1 — Supplementary file1 (DOCX 4235 KB) [file 10461_2024_4279_MOESM1_ESM.docx]
